# A multi-branched EMS mutant of *Isodon lophanthoides* var. *graciliflorus* exhibits significant differences in phytohormones and diterpenoids

**DOI:** 10.1186/s12870-026-08709-1

**Published:** 2026-04-23

**Authors:** Yongzhen Zou, Keyu Liang, Jingfeng Gu, Shuangshuang Zhao, Ruoting Zhan, Junmin Liu, Liping Wang

**Affiliations:** 1 Key Laboratory of Chinese Medicinal Resource from Lingnan, Ministry of Education, School of Pharmaceutical Sciences, Guangzhou University of Chinese Medicine, Guangzhou, Guangdong 510006 China; 2 Dept. of Pharmacy, Shenzhen Hospital of Beijing University of Chinese Medicine, Shenzhen, 518172 Guangdong China; 3 Guangdong Agribusiness Tropical Agriculture Institute Co.,Ltd., Guangzhou, Guangdong 511365 China; 4 Guangdong Yintian Agricultural Technology Co., Ltd., Yunfu, Guangdong 527300 China

**Keywords:** *Isodon lophanthoides* var. *graciliflorus*, Multi-branched, Ethyl methanesulfonate induction, Diterpenoid biosynthesis, Gibberellin

## Abstract

**Background:**

*Isodon lophanthoides* var. *graciliflorus* (Benth.) H. Hara, serves as one of the botanical sources for the traditional Chinese medicine “Xihuangcao”. Mixed germplasm resources and insufficient high-quality cultivars hinder its sustainable and high-quality development.

**Results:**

In this study, we generated a multi-branched mutant *B88*, induced by ethyl methanesulfonate (EMS). It exhibits a 36.78% increase in branching, and a 39.64% increase in plant height at maturity compared to the wild-type (WT) plants, implying high-yield potential. Although Simple Sequence Repeat (SSR) molecular markers revealed genetic variations, it retained the same chromosome number as the WT, indicating genetic stability and continuity. Transcriptomics revealed differentially expressed genes (DEGs) enriched in phytohormone biosynthesis and signal transduction pathways, and metabolomic identified 54 differentially accumulated metabolites (DAMs), mainly flavonoids and terpenoids. DEGs and DAMs were significantly enriched in diterpenoid biosynthesis, particularly the gibberellin (GA) pathway, with 4.79-fold higher GA content compared to the WT and three key genes (*ent-Copalyl Diphosphate Synthase*,* CPS; Gibberellin 20-oxidase*,* GA20ox; Gibberellin 2-oxidase*,* GA2ox*) showing significant differences, suggesting that GA may have a comprehensive regulatory effect on plant height, branching, and accumulation of bioactive compounds in *I. lophanthoides* var. *graciliflorus*.

**Conclusions:**

This study strongly supported the value of the breeding material-*B88* mutant, enriched the genetic diversity of *I. lophanthoides* var. *graciliflorus*, and revealed the potential of EMS mutagenesis in the improvement of medicinal plants, offering new insights for elucidating the effects of EMS mutagenesis on the growth regulation, quality improvement, and germplasm innovation of *I. lophanthoides* var. *graciliflorus.*

**Supplementary Information:**

The online version contains supplementary material available at 10.1186/s12870-026-08709-1.

## Background

*Isodon lophanthoides* var. *graciliflorus* (Benth.) H. Hara, a member of the Lamiaceae family and *Isodon* genus, is a perennial herb distributed in southern China (Guangxi, Guangdong, and Fujian provinces), India, Myanmar, Nepal and Vietnam [1]. The dried aerial parts (stems and leaves) have been used for the traditional Chinese medicine “Xihuangcao”. This species produces abundant secondary metabolites, particularly diterpenoids, flavonoids, and phenolic acids [2]. It is clinically used to treat conditions such as acute icteric hepatitis and enteritis [3, 4], and also consumed daily as a health-promoting beverage, such as tea, instant granules, and soup in southern China [2]. As a medicinal and edible plant resource, *I. lophanthoides* var. *graciliflorus* faces substantial clinical demand. However, being a cross-pollinating species that primarily reproduces by seed in nature, the plant exhibits considerable morphological variation due to both genetic diversity and environmental influences [5]. Currently, challenges such as the absence of elite cultivars, mixed germplasm resources, non-standardized seedling cultivation, and inconsistent yield and quality of raw materials have highlighted the urgent need for systematic germplasm evaluation and innovation to develop superior varieties for production. In this study, we performed a preliminary characterization of the multi-branched mutant *B88* to investigate the potential molecular and physiological mechanisms underlying its branching phenotype. The mutant holds promise as a novel elite genetic resource for the genetic improvement of *I. lophanthoides* var. *graciliflorus*.

In recent years, mutagenic breeding has gained increasing prominence in plant research, emerging as a significant approach for germplasm innovation [6]. Among various chemical mutagens, ethyl methanesulfonate (EMS) is the most widely utilized chemical mutagen [7]. Numerous studies have successfully applied EMS mutagenesis to various medicinal plants and crops in breeding. For example, EMS treatment yielded genotypes with enhanced pod characteristics and stable inheritance of *Lens culinaris* [8]; produced novel floral traits in ray florets of *Chrysanthemum morifolium* [9]; other successful applications include *Medicago sativa* [10], *Pinellia ternata* [11], and crops including sugarcane [12], wheat [13], tomato [14] and so on. Research indicates that EMS primarily induces point mutations, though it may also cause chromosomal deletions [15, 16]. Therefore, it is necessary to identify the mutant plants in combination with other methods and investigate their variation at other levels.

Recent advances in modern biotechnology have established RNA sequencing (RNA-seq) as a powerful tool for studying secondary metabolite biosynthesis pathways and their regulatory mechanisms, owing to its high throughput, sensitivity, and resolution [17, 18]. Metabolomics enables systematic detection and quantitative analysis of endogenous small-molecule metabolites in biological systems [19]. This high-throughput approach provides valuable insights into the complex relationships between metabolic pathways and their products [20]. At present, the integration of metabolomics and transcriptomics has emerged as an effective strategy for investigating secondary metabolite accumulation mechanisms and identifying key regulatory genes [21, 22]. This dual approach examines metabolic pathways from both causal (gene expression) and resultant (metabolite profile) perspectives, thereby elucidating the molecular basis of quality variations in medicinal plants [23]. It is widely used in medicinal plants, such as *Casuarina equisetifolia* [24], *Acanthopanax senticosus* [25], *Atractylodes macrocephala* Koidz [26], and *Curcuma* [27].

However, while transcriptome sequencing studies have been conducted on *I. lophanthoides* var. *graciliflorus* and related species [22, 28], these investigations have primarily focused on transcriptome data acquisition without exploring the combined application of transcriptomics and metabolomics in germplasm innovation and varietal selection. Based on this, we employed EMS mutagenesis to establish a preliminary mutant library, from which four elite mutants were selected from the M_2_ generation [5]. Notably, the multi-branched mutant demonstrates substantial yield potential due to its distinctive morphology and represents a promising new germplasm type. To validate whether it is capable of becoming a new germplasm type and to elucidate the underlying genetic and phenotypic changes, this study characterized the EMS-induced multi-branched mutant through comprehensive phenotypic analysis, chromosome identification, and SSR marker verification. Furthermore, integrated transcriptomic and metabolomic analyses revealed differential gene expression and metabolic regulation in hormone and diterpene biosynthesis pathways. These findings provide a foundation for developing new varieties from this multi-branched mutant and advancing breeding programs for *I. lophanthoides* var. *graciliflorus*.

## Materials and methods

### Plant materials

The EMS-induced multi-branched mutant selected from the M_2_ generation of *I. lophanthoides* var. *graciliflorus* was designated as *B88*. Both *B88* (M_2_ generation) and wild-type (WT) plants were propagated through cuttings and cultivated under standardized conditions. During the rapid growth phase two months post-transplantation, young buds were randomly collected and immediately preserved in a pretreatment solution for chromosome ploidy analysis. Concurrently, the third pair of leaves were harvested from randomly selected plants for subsequent transcriptomic and metabolomic analyses. All plant materials were maintained in the germplasm resource nursery at Guangzhou University of Chinese Medicine’s Shizhen Mountain facility, receiving uniform cultivation management including scheduled irrigation and fertilizer application.

### Observation of phenotype

Phenotype determination of M_2_ generation plants was conducted at maturity using standardized measurement protocols. Plant height was determined as the vertical distance from the rhizome base to the apical growth point of the main stem (including inflorescence) using a straightened plant specimen, measured with a tape measure. Stem diameter was measured 3**–**5 cm above the potting substrate using vernier calipers. Branches were quantified by counting all main stem branches containing ≥ 2 nodes. Leaf dimensions were assessed on the third pair of functional leaves: leaf length was measured along the midrib, while leaf width represented the maximum transverse distance, both determined using a precision ruler. The tape measure, vernier calipers and ruler were all purchased from Zhejiang Top Instrument Co., Ltd. Three biological replicates were set, and statistical significance was evaluated using SPSS 21.0 software.

### Chromosome ploidy identification

Through systematic optimization of pretreatment, dissociation, and staining conditions, we established an improved acidolysis protocol for chromosome preparation. Optimal conditions were determined as follows: fresh samples collected during peak mitotic activity (8:30**–**9:00 am) were pretreated with 0.002 mol·L^− 1^ 8-hydroxyquinoline for 2 h, followed by fixation in methanol: acetic acid (3:1) at 4 ℃ for ≥ 4 h. After rinsing with distilled water, cells were subjected to hypotonic treatment with 0.075 mol·L^− 1^ KCl for 20 min and subsequently digested in 1 mol·L^− 1^ HCl at 60 ℃ for 15 min. Following thorough washing, samples were stained with modified carbol fuchsin solution for 15 min. Following acid hydrolysis, specimens were subjected to three consecutive distilled water rinses, then stained with modified-carbol fuchsin solution for 15 min. Excess stain was removed by blotting with filter paper, followed by a final distilled water wash to eliminate floating dye. Chromosome spreads were examined under a Sunny EX30 light microscope (Sunny Optical Technology (Group) Co., Ltd., China) at 100× magnification. Thirty well-dispersed cells were analyzed per sample, applying the established criterion that ≥ 85% concordance in chromosome number indicates the species’ base chromosome count [29].

### SSR molecular markers identification

DNA was extracted from the specimens with the DNAsecure Plant Kit (DP320, China). DNA concentration and purity were evaluated using a NanoDrop 2000 ultramicro UV spectrophotometer (NanoDrop, ThermoFisher Scientific, USA), and quality was confirmed by 1% agarose gel electrophoresis. 24 polymorphic SSR primers (Table S1) of *I. lophanthoides* var. *graciliflorus* obtained through the previous screening were selected for polymorphism analysis. All primers were synthesized by Beijing Tsingke Biotech Co., Ltd. (Beijing, China). All PCR reactions were carried out using a T100™ Thermal Cycler PCR instrument (Bio-RED company, USA). The PCR products were detected with 8% polyacrylamide gel electrophoresis (PAGE). The SSR molecular marker identification method was based on that of Liu et al. [5].

### Transcriptome analysis

RNA was extracted from the specimens with the SteadyPure Plant RNA extraction kit (AG, China). The concentration of the extracted total RNA was measured with a NanoDrop ultra micro ultraviolet spectrophotometer (Thermo Fisher Scientific, United States) and the quality of the total RNA was detected with 1.0% agarose gel electrophoresis. The transcriptome sequencing was carried out by Guangzhou Gideo Biotechnology Co., Ltd. on an Illumina Nova-6000 platform. Fastp was used for quality control of raw reads, and low-quality data were filtered to obtain clean reads [30]. Trinity software was used for de novo assembly of clean reads [31]. The Unigene sequence was aligned to the protein database Non-redundant Protein (NR), Swiss Protein (Swiss-Prot), Kyoto Encyclopedia of Genes and Genomes (KEGG) and Clusters of Orthologous Groups (COG)/Eukaryotic Orthologous Groups (KOG) (E value < 0.00001) by BLASTx to obtain the protein with the highest sequence similarity for a given Unigene, thus yielding functional annotation information for the Unigenes. EdgeR software was used to normalize read counts, and the hypothesis test probability (*p*-value) was calculated according to the model [32]. Finally, multiple hypothesis testing correction was performed to obtain the False Discovery Rate (FDR). Genes with FDR < 0.05 and |log_2_FC| > 1 were screened as significantly differentially expressed genes, and KEGG enrichment analysis was performed to determine the most important biochemical metabolic pathways and signal transduction pathways in which differentially expressed genes (DEGs) were involved in.

### Quantitative real-time PCR (qRT-PCR)

Eight DEGs were selected for validation by quantitative real-time PCR (qRT-PCR). Primer sequences are provided in Supplementary Table S2. The qRT-PCR protocol followed previously established conditions from our research group, including the use of validated reference genes [33]. The expression stability of candidate reference genes was rigorously evaluated using four commonly used analytical methods, geNorm, NormFinder, BestKeeper, and the Delta CT method, and the results of these four methods were further integrated and comprehensively ranked using RefFinder. Specifically, the candidate reference genes evaluated included two categories: 59 reference genes (such as *C25*) screened based on leaf transcriptome sequencing data of *I. lophanthoides* var. *graciliflorus*, and 13 commonly used reference genes in plants (including *ACT*,* CDPK*,* EF-1α*,* GAPDH*, etc.). Based on the comprehensive stability ranking generated by RefFinder, *C25*, which showed the highest comprehensive stability among all candidate reference genes, was finally selected as the reference gene for subsequent experiments [33]. Three biological replicates per sample group, and each biological replicate was analyzed in triplicate technical repetitions.

### Metabolomics analysis by UPLC-MS/MS

The metabolomics analysis of the multi-branched mutant *B88* and the control groups was carried out by Guangzhou Gideo Biotechnology Co., Ltd. Six biological replicates were prepared for each sample. Analysis conditions were provided in the Document S1. Principal component analysis (PCA) was performed using the R gmodels package (v2.18.1). Partial least squares-discriminant analysis (PLS-DA) and Orthogonal partial least squares discriminant analysis (OPLS-DA) were implemented through the R ropls package. Significant differential metabolites were identified by combining OPLS-DA variable importance in projection (VIP) scores (VIP ≥ 1) with univariate t-test results (*p* < 0.05) [34]. These metabolites were subsequently Z-score normalized and subjected to hierarchical clustering analysis using the R pheatmap package (v1.0.12) for visualization. Pathway enrichment analysis was conducted by submitting the differential metabolites to the KEGG database (http://www.genome.jp/kegg/), with the significance threshold set at *p* < 0.05. Integrative analysis mapped both DEGs and differentially accumulated metabolites (DAMs) to KEGG pathways to identify shared metabolic pathways.

### Exogenous application of GA

Randomly selected WT plants with uniform and good growth conditions (30-day-old seedlings) were used as the experimental seedlings. GA (Lot: C16543124) and Paclobutrazol (PAC, GA biosynthesis inhibitor, CAS: 76738-62-0) were dissolved in ethanol (0.1 M) and diluted with ddH_2_O to a concentration of 500 µM. Plants were first sprayed at 14 days after planting, and then sprayed once a day for a total of three consecutive days. Control plants (CK) were sprayed with a solution without GA or PAC. Plant height, main stem primary branches, main stem diameter and nodes were measured at 9 and 21 days after treatment (*n* = 10). The measurement methods for plant height, main stem primary branches and main stem diameter were described in Sect. 2.2, and nodes number refers to the total number of nodes on the main stem.

## Results

### Mutant *B88* exhibits increased plant height and branching with chromosome stability and SSR differences

**Fig. 1:**
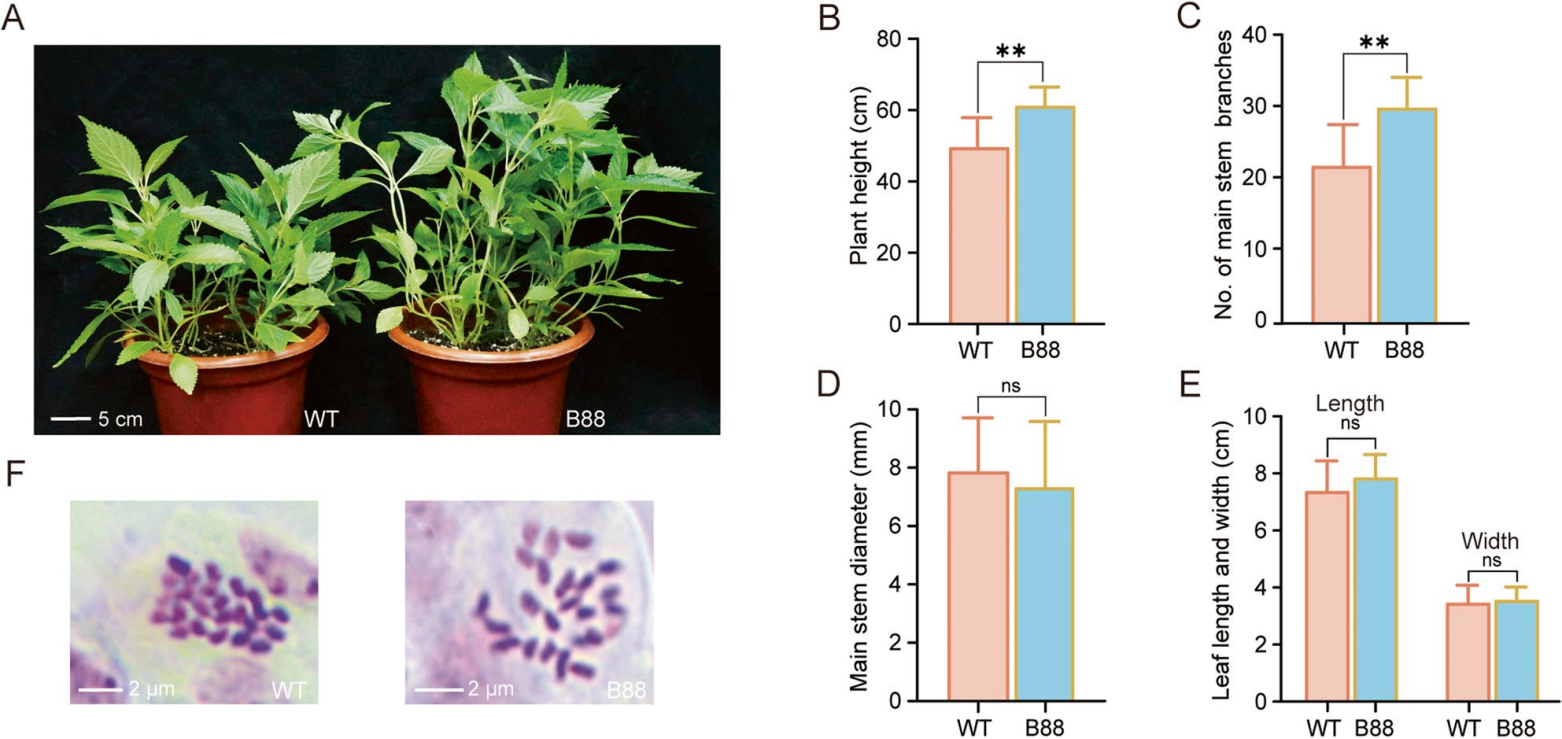
Plant characteristics and chromosome ploidy of *B88* mutant of *Isodon lophanthoides* var. *graciliflorus* (Benth.) H. Hara. **A** Morphological characteristics of the transplanted plants two months after planting. **B**–**E** Phenotypic traits of the transplanted plants at four months. Significant differences were observed in the plant height (**B**) and the number of main stem primary branches (**C**), whereas no significant differences were found in the main stem diameter (**D**), leaf length and leaf width (**E**). Data are presented as mean ± SD (*n* = 20), “*” indicates *p* < 0.05, “**” indicates *p* < 0.01. **F** Identification of chromosome ploidy: No differences in metaphase chromosome morphology were observed between *B88* and WT under a light microscope. Full-length blots are provided in Additional File 1

In order to explore the differences between WT and mutant *B88*, we observed the growth of M_2_ generation *B88* and WT (Fig. 1A), While no significant variations were observed in main stem diameter (Fig. 1D), leaf length or width (Fig. 1E), the *B88* mutant exhibited pronounced enhancements in two key growth parameters: a 36.78% increase in the number of main stem branches (Fig. 1C) and a 39.64% increase in plant height (Fig. 1B) compared to WT controls. These findings suggested that EMS mutagenesis may have enhanced both vertical growth potential and branching capacity in the aerial portions of *B88* plants.

Meanwhile, chromosome identification revealed that mutant *B88* maintained the same diploid chromosome number (2n = 24) as WT plants (Fig. 1F). During interphase, uniformly stained nuclei showed no visible chromosomes (Fig. S1A); prophase exhibited chromatin condensation into discernible chromosomes (Fig. S1B); metaphase displayed highly condensed and distinct chromosomes, which was the ideal stage for chromosome analysis (Fig. S1C); during anaphase, chromosomal separation occurred at centromeres, with spindle fibers facilitating the orderly migration of sister chromatids to opposite poles, ensuring equal distribution of genetic material to daughter cells (Fig. S1D). In telophase, following poleward chromosome segregation, cells restore the nucleolus and reestablish the nuclear envelope as chromosomes progressively decondense from their highly condensed state, dispersing throughout the nascent nuclear compartment (Fig. S1E). Therefore, the chromosome number of mutant *B88* and WT were observed in the metaphase (Fig. S1C), and the results showed that the chromosome number of *B88* was the same as that of WT, which was 24 chromosomes (Fig. 1F).

However, specificity was successfully detected in *B88* mutants, even though the chromosome number remained unchanged. Among 314 loci amplified by 24 polymorphic SSR primers, 146 (42.43%) exhibited polymorphism (Table S3, Fig. S2). Primer 17 showed the highest polymorphism rate (92.86%), followed by primer 19 (90.91%). Primer 23 produced the fewest amplification products (5 loci) and primer 13 generated the most (23 loci) (Table S3, Fig. S2). On average, each primer pair amplified 13.08 loci, with 6.08 being polymorphic (Table S3, Fig. S2). This variation in amplification likely reflects differential distribution of genetic diversity across genomic regions.

### RNA sequencing and transcriptomic characteristics of mutant *B88*

**Fig. 2:**
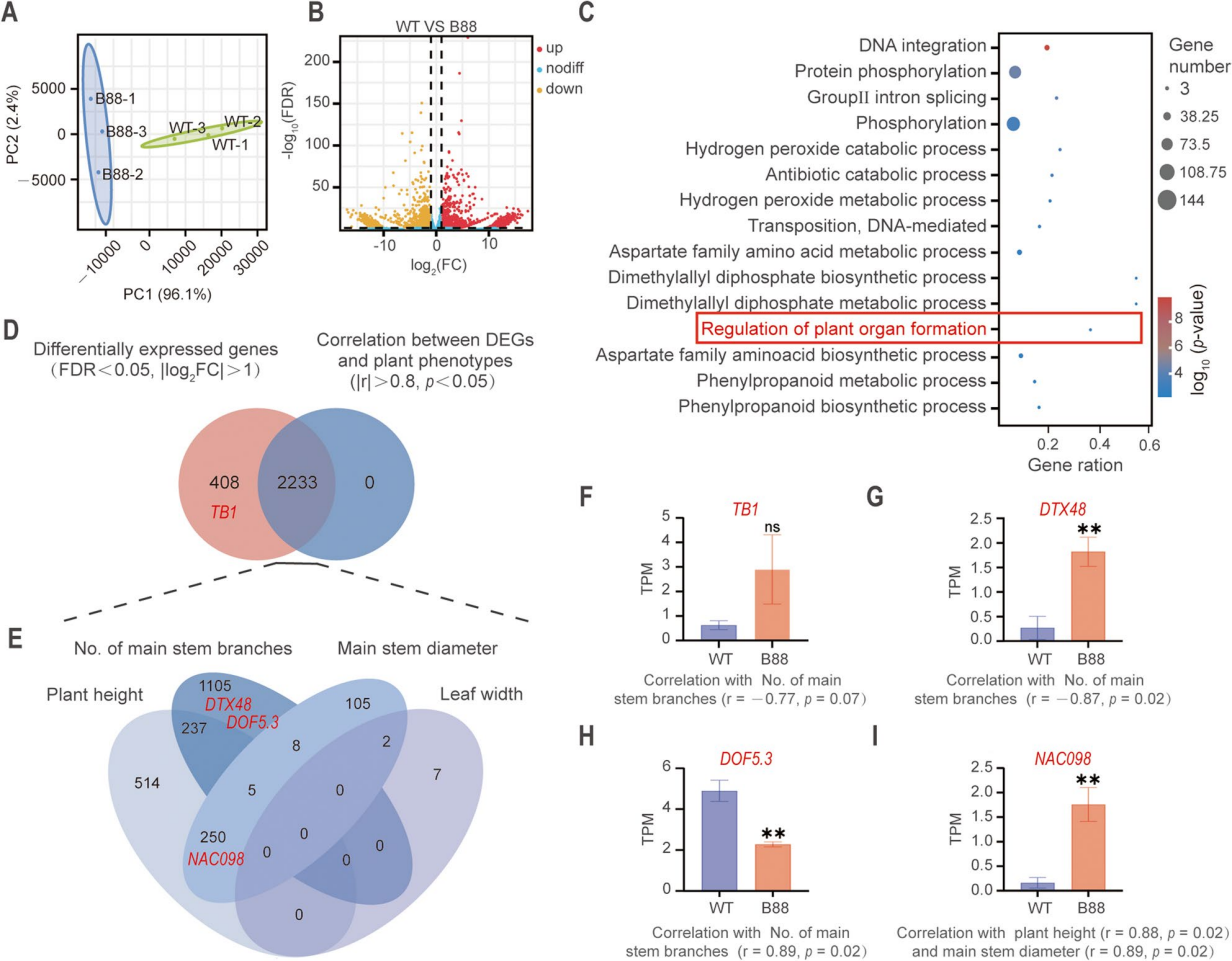
Gene expression changes in RNA-seq of *B88* mutant in *I. lophanthoides* var. *graciliflorus. ***A **Principal Component Analysis (PCA) demonstrating high reproducibility among replicates and clear separation between WT and *B88*. **B **Volcano plot showing up-regulated and down-regulated differentially expressed genes (DEGs) in WT vs. *B88*. Each dot in the volcano plot represents an individual gene, and the closer to the genes at either end, the greater the degree of difference. **C **Bubble plot of Gene Ontology (GO) enrichment analysis of Biological Process (BP) terms. DEGs were significantly enriched in the Regulation of plant organ formation (GO:1905428) under the Developmental process (GO: 0032502). **D **Venn diagram illustrating the overlap between all DEGs and phenotype-associated DEGs (|r| > 0.8, *p* < 0.05), identifying 2233 overlapping DEGs. **E **Distribution of the overlapping DEGs across five phenotypic traits, with 1006, 1355, 310, 0, and 9 DEGs were associated with plant height, branching, stem diameter, leaf length, and leaf width, respectively. **F–I **Transcripts Per Million (TPM) values and correlation coefficients for four key DEGs associated with the GO term (Regulation of plant organ formation)

To explore potential mechanisms underlying the multi-branched phenotype in *I. lophanthoides* var. *graciliflorus* mutants, we performed transcriptomic analysis of leaf samples from mutant *B88* and WT plants. High-throughput sequencing generated 41**–**52 million raw reads per sample, with over 99.73% high-quality clean reads following quality control, with GC content of 48.08%**–**48.58% and Q30 scores exceeding 95.53% (Table S4). These metrics suggest that the RNA-seq experiments yielded high-quality transcriptomic data. Annotation against multiple databases (KOG, NR, Swiss-Prot and KEGG) identified a total of 24,707 unique genes. Validation experiments using qRT-PCR on eight selected DEGs involved in hormone biosynthesis and signaling pathways, suggested expression trends consistent with RNA-seq results (Fig. S3). PCA revealed strong intra-group reproducibility and indicated clear inter-group separation (Fig. 2A). Comparative analysis identified 2,641 DEGs in the WT vs. *B88*, comprising 1,113 upregulated and 1,528 downregulated genes (Fig. 2B, Table S5).

Gene Ontology (GO) analysis classified the DEGs into three primary categories: biological process (BP), molecular function (MF), and cellular component (CC), with further annotation into 42 secondary classifications. The largest quantity DEGs enriched in BP terms were cellular process (GO:0009987), while the predominant MF terms were binding (GO:0005488). CC terms showed relatively lower DEGs enrichment, with cellular anatomical entity (GO:0110165) and protein-containing complex (GO:0032991) being most prominent (Table S5). Using the *p*-value corrected by FDR<0.05 as the criterion for screening significant enriched terms. Notably, the developmental process (GO:0032502) exhibited significant DEGs enrichment (Fig. 2C), particularly involving four genes associated with plant organ formation regulation (GO:1905428) (Fig. 2C): *TB1* (hypothetical protein, *Unigene0009056*), *DOF5.3* (Dof zinc finger protein *DOF3.1*, *Unigene0012885*), *DTX48* (MATE efflux family protein, *Unigene0022086*), and *NAC098* (NAC domain transcriptional regulator, *Unigene0022382*). *TB1*, *DTX48* and *NAC098* showing upregulation and *DOF5.3* demonstrating downregulation (Fig. 2D–I). Through correlation analysis, 2233 DEGs with high correlation to the phenotype were obtained (|r| > 0.8, *p* < 0.05) (Fig. 2D), including 1006, 1355, 310, 0, and 9 DEGs associated with plant height, the number of main stem branches, main stem diameter, leaf length, and leaf width, respectively (Fig. 2E). Among them, the number of DEGs highly correlated with main stem branches was the largest, followed by those related to plant height (Fig. 2E). *DTX48* and *DOF5.3* were highly correlated with main stem branches (Fig. 2G–H); *NAC098* was highly correlated with both plant height and main stem diameter (Fig. 2I); *TB1* showed a correlation with main stem branches, but the correlation was not significant (Fig. 2F). Overexpression of *DTX48* may attenuate apical dominance [35] (Fig. 2G). *DOF5.3* can activate the expression of genes related to the radial growth of protophloem sieve elements, promoting vascular bundle organization and cell differentiation; its downregulated expression may reduce the promotive effect on cell differentiation [36] (Fig. 2H). *NAC098* is involved in the formation of the shoot apical meristem (SAM), mainly restricting growth by inhibiting cell division, and its upregulated expression may enhance the inhibition of cell division [37] (Fig. 2I). The core function of *TB1* is to repress lateral branch growth, thereby maintaining apical dominance, and its upregulated expression may strengthen apical dominance in plants [38] (Fig. 2F). The significant upregulation or downregulation of these 4 genes appears to coordinately modulate plant growth and development.

### Hormone signals may comprehensively coordinate the branched phenotype and metabolite levels of *I. lophanthoides* var. *graciliflorus*

**Fig. 3:**
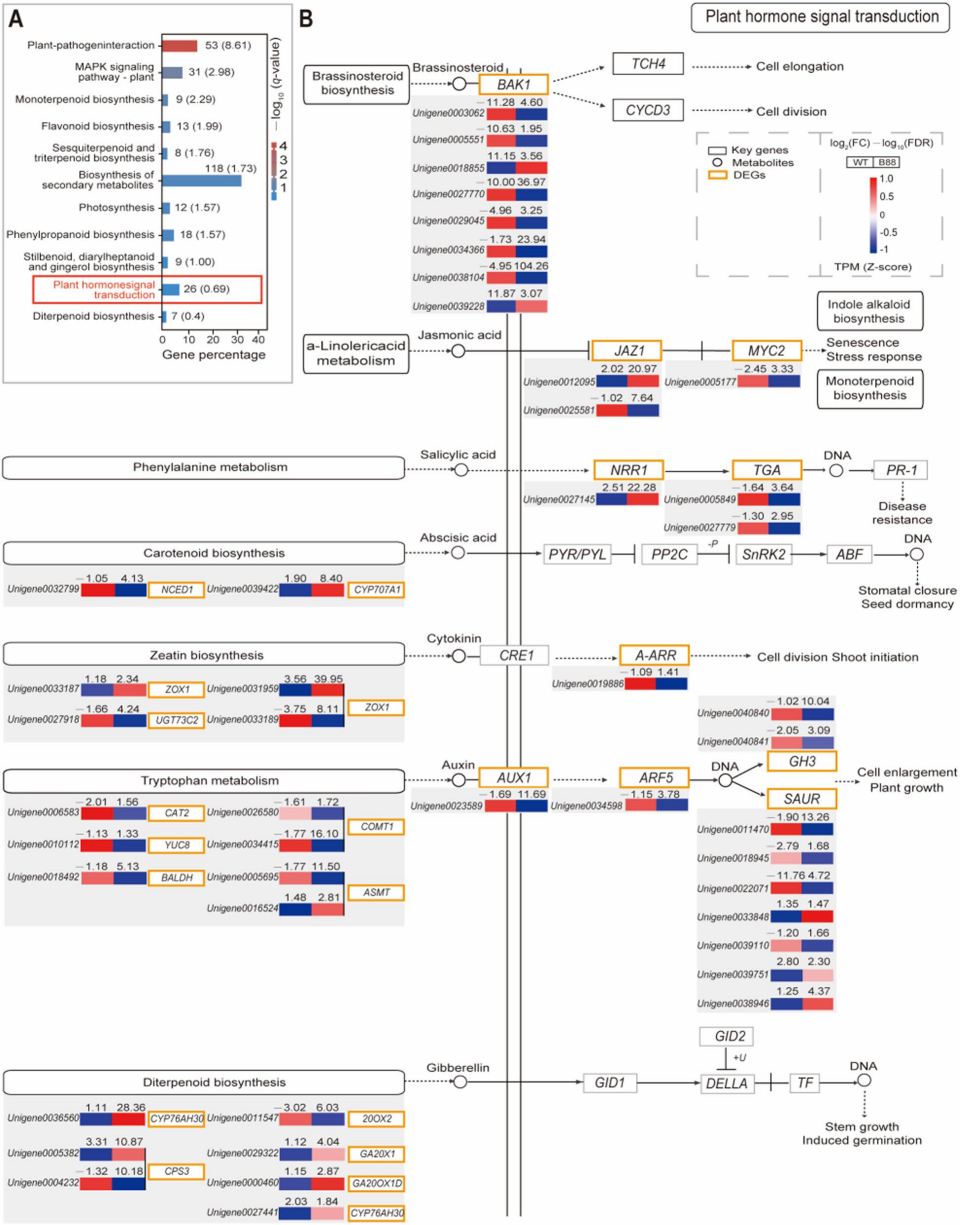
Expression levels of DEGs involved in key hormone synthesis and signal transduction significant pathways. **A **Top 11 significantly enriched KEGG pathways for the DEGs. **B** Synthetic path diagram of Plant hormone signal transduction (ko04075). The heatmap illustrates the expression profiles of DEGs related to hormone biosynthesis and signal transduction (TPM, Z-score standardized). Genes displayed in the boxes represent annotation information of KEGG database

KEGG enrichment analysis of DEGs identified 11 significantly enriched metabolic pathways (Fig. 3A). Among these, the plant hormone signal transduction pathway (ko04075) showed substantial transcriptional changes (Fig. 3A). Additionally, secondary metabolic pathways—including those for diterpenoid, monoterpenoid, sesquiterpenoid, triterpenoid, and flavonoid biosynthesis—were also significantly enriched (Fig. 3A). These secondary metabolic processes were regulated by hormone signals; conversely, the altered flux of secondary metabolism may indirectly modulate hormone homeostasis via feedback regulation or competition for shared precursor metabolites. For instance, gibberellins (GAs) are synthesized via the diterpenoid pathway [39], while specific flavonoids may serve as hormonal precursors or modulators, potentially influencing plant growth and development through their effects on hormone biosynthesis and homeostasis. In the meanwhile, the differential expression of flavonoid and diterpenoid biosynthesis pathways may also affect the formation of secondary metabolites in *I. lophanthoides* var. *graciliflorus*, which may affect the quality of medicinal materials.

To explore potential molecular mechanisms underlying the multi-branched phenotype in *I. lophanthoides* var. *graciliflorus*, we conducted detailed analysis of DEGs associated with plant hormone signal transduction (ko04075) and related biosynthesis pathways (Fig. 3B). Transcriptomic profiling identified 26 DEGs related to hormone signal transduction (Fig. 3A), with only 7 showing upregulation while 19 were downregulated (Fig. 3B). The auxin (Indole-3-Acetic Acid, IAA) signaling pathway contained the highest number of DEGs (*n* = 11), spanning the *AUXin1/Like-AUX (AUX/LAX)*,* Auxin Response Factor (ARF)*, *Gretchen Hagen 3 (GH3)*, and *Small Auxin-Up RNA (SAUR)* gene families, including *AUX1 (AUX1 LAX family)*, *ARF5 (ARF family)*, *CH3.1 (CH3 gene family)*, *ARG7 (SAUR family)*, *SAUR20 (SAUR family)*, *SAUR23 (SAUR family)*, *SAUR32 (SAUR family)*, with only *ARG7*, *SAUR20*, and *SAUR23* demonstrating upregulated expression in *B88* mutants. The cytokinin (CTK) signaling transduction exhibited one downregulated DEG *(Response Regulator 10*,* RR10)*, while brassinosteroid (BR) signaling transduction involved 7 DEGs in the *BR-Associated Kinase 1 (BAK1) family* (5 downregulated, 2 upregulated). Jasmonic acid (JA) signaling transduction included 3 DEGs (2 *Jasmonate Zim-Domain*,* JAZ)* genes with opposing regulation patterns and 1 downregulated *MYC-like transcription factor 2 (MYC2)*, and salicylic acid (SA) signaling transduction contained 3 downregulated DEGs (1* Negative Regulator of Resistance 1*,* NRR1* and 2 *Transcription factor with G-box Association*,* TGA* genes) (Fig. 3B).

Integrated analysis of transcriptomic data and KEGG pathway enrichment identified DEGs associated with the biosynthesis of key phytohormones including IAA, GA, abscisic acid (ABA), and CTK (Fig. 3B). The expression profiles of key genes involved in IAA, CK, ABA, and BR biosynthesis and signaling pathways (e.g., *YUC8*,* ZOX1*,* NCED1*,* BAK1*) exhibited changes in the *B88* mutant compared with the wild type (Fig. 3B). The synthesis of IAA mainly occurs in the indole pyruvate pathway (IPA) of Tryptophan metabolism (ko00380). In plants, tryptophan is converted to indole pyruvate through a series of enzymatic reactions, which ultimately produce IAA [40]. In Tryptophan metabolism (ko00380) *YUCCA 8 (YUC8)* and *Benzaldehyde Dehydrogenase (BLADH)* were down-regulated (Fig. 3B). In Tryptophan metabolism (ko00380) *YUC8* and *BLADH*, were down-regulated, which may indicate the block of auxin synthesis in the multi-branched mutant (Fig. 3B). Diterpenoid biosynthesis (ko00904) analysis revealed upregulated *Gibberellin 2-oxidase 1 (GA2ox1)* and *Gibberellin 20-oxidase 1D (GA20ox1D)* alongside downregulated *Gibberellin 20-oxidase 2 (GA20ox2)* in GA synthesis (Fig. 3B). Carotenoid biosynthesis (ko00906) pathway analysis demonstrated upregulated *CYPtochrome P450 family 707 subfamily A polypeptide 1 (CYP707A1)* and downregulated *9-Cis-Epoxycarotenoid Dioxygenase 1 (NCED1)* in ABA metabolism (Fig. 3B). Zeatin biosynthesis (ko00908) screening identified one downregulated *UDP-glycosyltransferase 73C2 (UGT73C2)* and three *Zeatin O-glucosyltransferase-like protein 1 (ZOX1)* genes (two downregulated, one upregulated) potentially involved in CTK regulation (Fig. 3B). These hormone-related transcriptional alterations appear to contribute to the branching phenotype of *I. lophanthoides* var. *graciliflorus*, providing valuable insights for future investigations of branching regulation mechanisms in this species.

### Overview of the metabolic profiles of *I. lophanthoides* var. *graciliflorus B88*

**Fig. 4:**
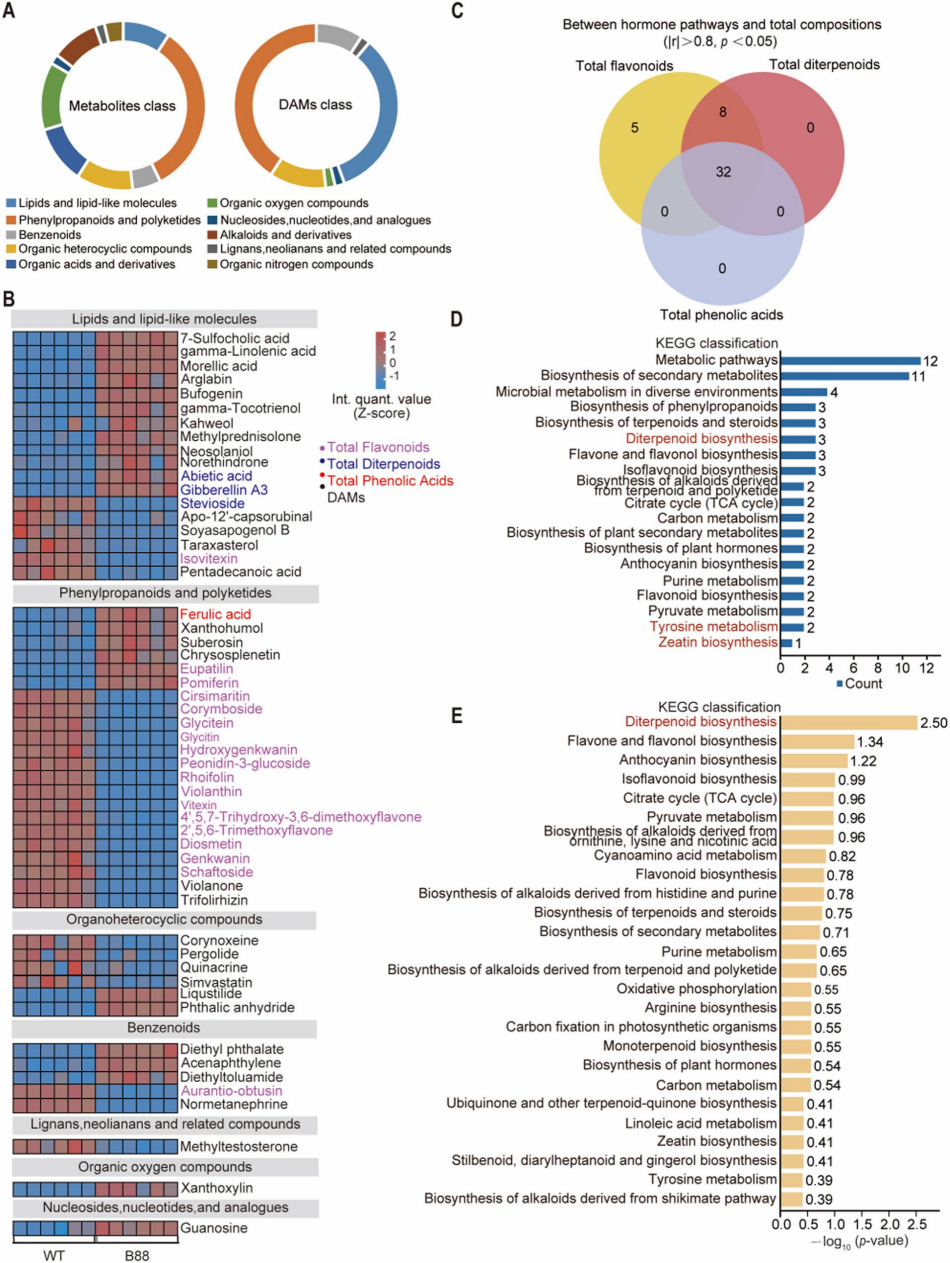
Metabolomic analysis results of the *B88* mutant. **A **Proportions of identified metabolites across chemical categories (left) and classification of differentially accumulated metabolites (DAMs) (right). **B **Hierarchical clustering analysis of the DAMs. Highlighted the DAMs, corresponding to the quality indicators of *I. lophanthoides* var. *graciliflorus*, including total diterpenoids, total flavonoids and total phenolic acids. **C **Correlation analysis between these DAMs and DEGs involved in hormone pathways (|r| > 0.8, *p* < 0.05). **D–E **KEGG pathway enrichment analysis of the DAMs, DAMs were selected based on the following criteria: *p*-value < 0.05, fold change (FC) ≤ 0.5 or ≥ 2, VIP ≥ 1. (D) Top 19 KEGG pathways containing the highest number of DAMs. **E **Top 26 significantly enriched KEGG pathways for the DAMs

Comprehensive metabolic profiling of *B88* and WT of *I. lophanthoides* var. *graciliflorus* identified and quantified 437 metabolites through combined positive and negative ion mode analyses (Table S6). PCA (Fig. S4A**–**B) and hierarchical clustering (Fig. 4B) showed significant differences in the metabolite components between the six *B88* samples and WT samples. OPLS-DA further supported these metabolic differences, showing excellent model reliability and clear group separation in score plots (Fig. S4C**–**F). According to metabolite classification, lipids and lipid-like molecules represented the most abundant class (35.2%), including lignan lipids, steroids, steroid derivatives and fatty acids (Fig. 4A). Phenylpropanoids and polyketides (15.6%) were the second most abundant group, comprising flavonoids, isoflavones and cinnamic acid derivatives (Fig. 4A). OPLS-DA identified 54 significant DAMs, accounting for 12.37% of all metabolites. Phenylpropanoids and polyketides, together with Lipids and lips-like molecules, were the most abundant classes, whereas alkaloids and derivatives were less represented (Table S7). Among these DAMs, 29 were downregulated (notably violanone among phenylpropanoids and polyketides) and 25 upregulated (particularly neosolaniol among organic oxygen compounds) (Fig. 4B). Furthermore, among the 54 DAMs, 22 of them contain 18 total flavonoids, 3 total diterpenoids and 1 total phenolic acid, which are the main active ingredients of *I. lophanthoides* var. *graciliflorus* (Fig. 4C). Among the total flavonoids, only 2 were upregulated, while the remaining 16 were downregulated. This may imply that the *B88* mutant, despite its potential for high yield, could exhibit a reduction in total flavonoid secondary metabolites content. Correlation analysis was performed between these DAMs and DEGs involved in hormone pathways, and 45 DEGs showed significant correlations with the DAMs of the total components (|r| > 0.8, *p* < 0.05) (Fig. 4C). Among them, 32 DEGs involved in hormone pathways showed significant correlations with all three total components, 5 DEGs were significantly correlated only with total flavonoids, and 8 DEGs exhibited significant correlations with both total flavonoids and total diterpenoids (|r| > 0.8, *p* < 0.05) (Fig. 4C).

KEGG pathway enrichment analysis of DAMs revealed 55 metabolites mapping to 39 metabolic pathways (Table S8). The most abundant DAMs participating in Metabolic pathways, Biosynthesis of secondary metabolites, Microbial metabolism in diverse environments, and Diterpenoid biosynthesis (descending order) (Fig. 4D). The diterpenoid biosynthesis pathway emerged as the most significantly enriched (Fig. 4E). In addition, DAMs were also significantly enriched in flavone and flavonol biosynthesis, isoflavonoid biosynthesis, tyrosine metabolism and Zeatin biosynthesis (Fig. 4D–E). Tryptophan is a key precursor for auxin synthesis [41]. Zeatin is an active form of cytokinin [42]. These findings were consistent with the transcriptome sequencing results. Integrated transcriptome and metabolome analysis suggested that the altered gene expression in the hormone metabolic pathway of mutant *B88*, affected metabolite accumulation, ultimately modifying growth and developmental. These developmental changes may be associated with a substantial depletion of other secondary metabolites.

### Combined analyses transcriptomic and metabolomic

**Fig. 5:**
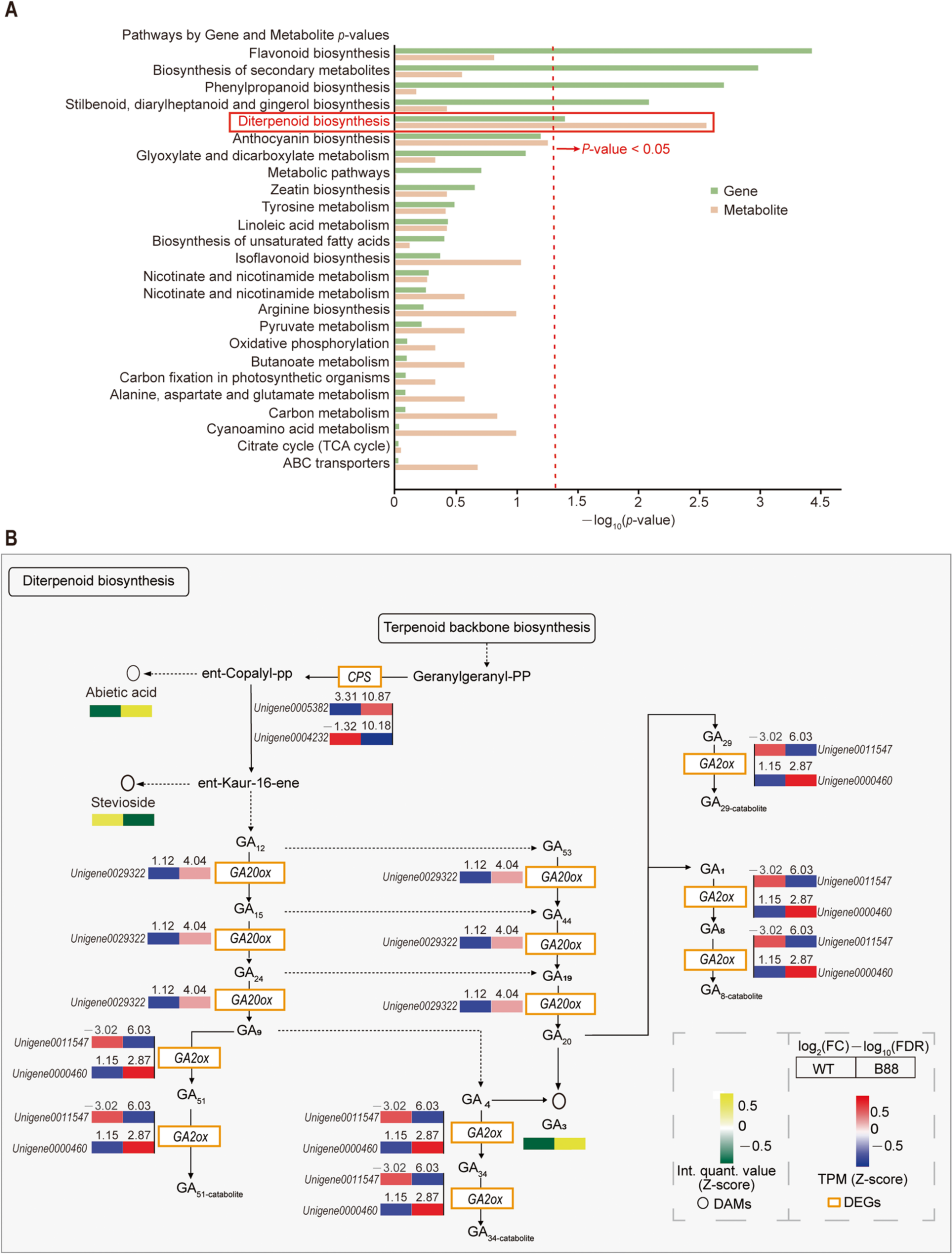
Integrated transcriptomic and metabolomic analysis. **A **KEGG enrichment analysis. Using a significance threshold of *p* < 0.05, only diterpenoid biosynthesis pathways was significantly co-enriched, indicating that both DEGs and DAMs within this pathway differed significantly between WT and *B88*. **B **Detailed analysis of the diterpenoid biosynthesis pathway. DAMs in this pathway included abietic acid, stevioside, and gibberellin A_3_ (GA_3_). Compared to the WT, the accumulation levels of abietic acid and GA_3_ increased, whereas stevioside was decreased in the *B88* mutant. DEGs encoding key enzymes involved in diterpenoid biosynthesis included *CPS*, *GA20ox*, and *GA2ox*. Gene names in boxes indicate KEGG database annotations, whereas those in parentheses represent Swiss-Prot annotations. The heatmap displays the relative gene expression and metabolite accumulation based on Z-score standardized TPM values and quantitative signal values

Integrated analysis of transcriptomic and metabolomic data demonstrated co-enrichment of DEGs and DAMs in diterpenoid biosynthesis pathways (Fig. 5A). The DEGs and DAMs were simultaneously mapped to the KEGG pathway database to obtain the relevant information of the common pathway (Fig. 5B). The DEGs and DAMs were mainly enriched in Diterpenoid biosynthesis (ko00904), especially the synthetic pathway of the diterpenoid compound gibberellin showed significant differences. The analyses revealed the active component Gibberellin A_3_ (GA_3_) was up-regulated in the multi-branched mutant *B88* (Fig. 5B), which showing 4.79-fold higher compared to the WT. Two key genes encoding enzyme gene *CPS*, *Unigene0004232 (CPS3)*, were down-regulated and *Unigene0005382 (CPS3)* was up-regulated. Two genes encoding GA20ox, *Unigene0000460 (GA20ox1D)*, were down-regulated and *Unigene0011547 (20ox2)* was up-regulated. *Unigene0029322 (GA2ox1)*, the gene encoding GA2ox, was up-regulated (Fig. 5B). These DEGs were key genes for the synthesis of diterpenoids such as GAs, and play an important role in the process of GAs biosynthesis [43].

**Fig. 6:**
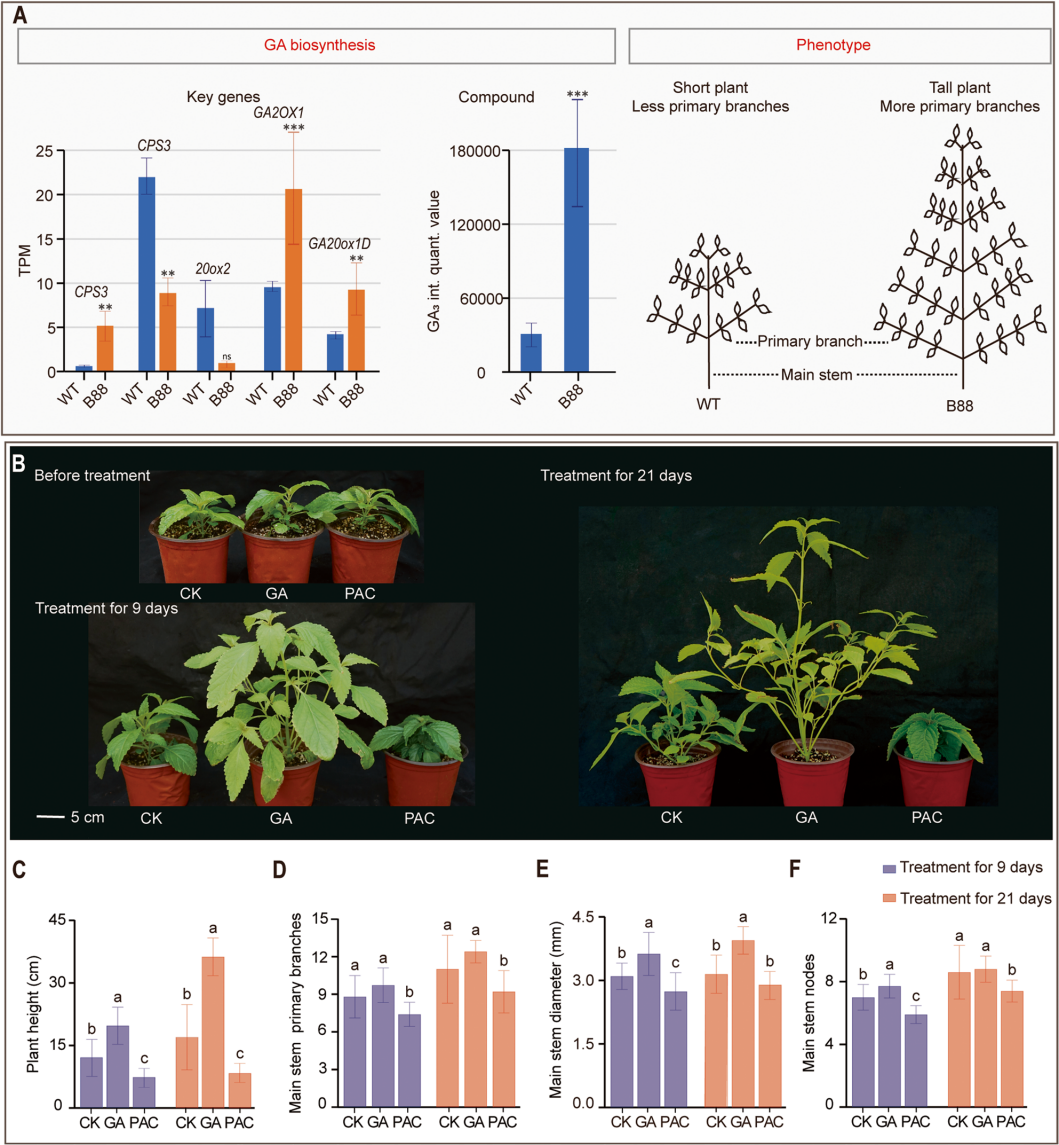
Exogenous GA verification and Potential mechanistic model of EMS-induced multi-branched mutants in *I. lophanthoides* var. *graciliflorus. ***A **Potential mechanistic model underlying the regulation of the multi‑branched phenotype by the GA pathway. The altered expression of key genes in the GA biosynthesis pathway affects endogenous GA levels, thereby regulating plant height and primary branch number of *I. lophanthoides* var. *graciliflorus*. These key genes include *CPS3* (*Unigene0004232, Unigene0005382*), *20ox2* (*Unigene0011547*), *GA20ox1D* (*Unigene0000460*), *and GA20ox1* (*Unigene0029322*). **B–F **The exogenous GA_3_/PAC experiment. Based on 30 days old wild type (WT) seedlings, 500 μM GA₃ or PAC was sprayed for three consecutive days, with untreated plants serving as controls. Plant phenotype (**B**) and phenotypic traits including plant height (**C**), primary branch number (**D**), main stem diameter (**E**), and node number (**F**) were measured at 9 and 21 days post treatment. Different lowercase letters (a, b, c) indicate significant differences (*p* < 0.05)

Consistent with previous phenotypic observations, mutant B88 displayed significantly higher main stem branch number and plant height than the WT. Further integrated transcriptome and metabolome analyses revealed that DEGs and DAMs significantly enriched in the diterpenoid biosynthesis pathway in KEGG database, especially the GA biosynthetic pathway. Differential expression was observed for *CPS*, *GA2ox* and *GA20ox* in this pathway. In the WT, the genes encoding CPS were *Unigene0004232 (CPS3)* and *Unigene0005382 (CPS3)*. The genes encoding GA2ox were *Unigene0000460 (GA20ox1D)* and *Unigene0011547 (20ox2)*, and the genes encoding GA20ox were *Unigene0029322 (GA20ox1)*. The Transcripts Per Million (TPM) values of these genes were 0.5157, 21.96, 4.135, 7.110, 9.413 in WT, and 5.105, 8.784, 9.176, 0.8740, 20.59 in mutant *B88*, respectively. The differential expression of these genes coordinately modulated GA biosynthesis, leading to significant differences in GA_3_ between WT and mutant *B88* (Fig. 6A). It was speculated that the variation in GA_3_ may be a key factor contributing to the phenotypic differences in mutant *B88*.

To further verify the transcriptomics and metabolomics results, we treated WT plants with exogenous GA and the GA biosynthesis inhibitor PAC (Fig. 6B). The results showed that exogenous GA application promoted plant height, branch number, stem diameter and node number to different degrees, whereas PAC treatment exerted the opposite effect (Fig. 6B–F). Compared with the CK group, GA treatment for 9 days increased plant height, branch number, stem diameter and node number by 63.03%, 10.39%, 17.08% and 10.20% respectively, with significant differences observed in plant height, stem diameter and node number. After 21 days of GA treatment, these traits increased by 113.66%, 12.73%, 25.39% and 2.33% respectively, with significant differences in plant height and stem diameter. In contrast, PAC treatment for 9 days decreased these traits by 38.98%, 15.91%, 11.59% and 15.71%, respectively, and by 50.50%, 16.36%, 8.01% and 13.95% after 21 days. At both 9 and 21 days after treatment, all four phenotypic traits differed significantly between the PAC and GA treatment groups (Fig. 6B–F).

## Discussion

This study used integrated transcriptomic and metabolomic analyses to explore the molecular basis underlying the phenotypic differences between the multi-branched mutant *B88* and WT controls of *I. lophanthoides* var. *graciliflorus*. The *B88* mutant exhibited significantly enhanced phenotypic characteristics, including a 36.78% increase in main stem branches and 39.64% greater plant height compared to the WT (Fig. 1B–C). Molecular characterization via SSR markers supported the mutant’s specificity (Fig. S2), while maintaining a diploid chromosome number (2n = 24) (Fig. 1F). Transcriptomic analysis revealed substantial alterations in hormone biosynthesis and signaling pathways (Fig. 3), particularly involving auxin and GA metabolism and so on. Metabolomic profiling identified flavonoids and terpenoids as the predominant DAMs (Fig. 4A–C), with the majority of flavonoid metabolites downregulated. DEGs involved in hormone pathways showed significant correlations with these DAMs associated with the total components (Fig. 4C). Terpenoids were notably enriched in the diterpenoid biosynthesis pathway (ko00904), specifically in GA biosynthesis pathway (Fig. 5). These findings provide crucial insights into the molecular mechanisms governing multi-branched and secondary metabolism in *I. lophanthoides* var. *graciliflorus*, establishing a foundation for targeted genetic improvement and quality enhancement of this species.

In plant breeding, EMS-induced mutagenesis has emerged as an efficient technique for germplasm innovation and improvement. Molecular marker analysis offers more direct and reliable genomic characterization than phenotypic evaluation, as demonstrated by our SSR genotyping using 24 primer pairs which detected a polymorphism ratio of 42.43% (Table S2). This indicates that EMS mutagenesis induces specific genetic modifications underlying phenotypic variation, while retaining most of the original genetic characteristics. Chromosomes serve as fundamental carriers of genetic information whose quantitative and structural alterations directly influence plant genetic characteristics and phenotypic expression. Cytogenetic analysis through chromosome ploidy identification provides critical insights into genetic stability and breeding potential of mutant plant (Fig. 1F). In this study, we employed the acid hydrolysis method due to its advantages of cost-effectiveness, procedural simplicity, time efficiency, broad applicability, and preparation quality stability. It was found that the chromosome ploidy of the multi-branched mutant *B88* produced by EMS mutagenesis did not change, both the *B88* and the WT control were diploid, consistent with the research results that the base chromosome of *Isodon* plants was x = 12 (Fig. 1F) [44]. The consistency of chromosomal ploidy and number suggested that *B88* maintains the genetic quantity and genomic structure specific to the WT. It also suggested that *B88* can retain relatively stable morphological, physiological and ecological characteristics during reproduction, thereby ensuring the stability and continuity of the species [45].

The EMS-induced multi-branched mutant *B88* of *I. lophanthoides* var. *graciliflorus* exhibited significant phenotypic alterations in plant height and branching while maintaining diploid stability (Fig. 1B–C). Correlation analysis revealed that 84.55% of the DEGs were significantly correlated with the four phenotypic traits (Fig. 2D). These DEGs may affect the multi-branched phenotype of the mutant by regulating key metabolic pathways, cell signal transduction and gene expression, particularly in plant hormone biosynthesis pathways and signaling pathways (Fig. 3).

On the one hand, DEGs were significantly enriched in plant hormone biosynthesis pathways of key phytohormones including *IAA*,* GA*,* ABA* and *CTK* (Fig. 3B). Notably, the IAA biosynthetic genes *YUC8* (Log_2_FC = －1.13) and *BALDH* (Log_2_FC = －1.18) showed marked downregulation. YUC-encoded flavin monooxygenases were core enzymes in auxin production [46], and *ALDH* family members participated in auxin metabolism [47]. This transcriptional suppression likely impairs IAA biosynthesis, potentially reducing endogenous auxin levels and disrupting the hormone’s critical roles in apical dominance maintenance and lateral bud suppression (Fig. 3B). The observed downregulation of *YUC8* and *BALDH* may therefore contribute to the multi-branched phenotype by attenuating auxin-mediated apical control and promoting lateral shoot development. However, branch formation is a complex developmental process involving multiple hormonal interactions; GA, ABA and CTK, pathways showed both up-regulated and down-regulated differential gene expression in this study, implying potential crosstalk among these phytohormones in regulating the observed phenotypic traits (Fig. 3B).

On the other hand, DEGs were significantly enriched in plant hormone signaling pathways, including IAA, BR, JA, and CTK (Fig. 3B). In auxin signaling transduction, *AUX1* (Log_2_FC = －1.69) is involved in the polar transport and distribution of auxin [48], *ARF* (Log_2_FC = －1.15) can bind to auxin response elements and regulate the expression of auxin response genes [49], *GH3* gene is involved in the metabolism and inactivation of auxin [50], *SAUR* may interact with components of auxin sensing and response to regulate the effects of auxin [51]. These differentially expressed genes were mainly down-regulated, suggesting IAA signal transduction network may be inhibited, leading to the impaired apical dominance in the multi-branched mutants (Fig. 3B). This inhibition may provide more growth opportunities for lateral buds and promoted branch development of the mutant strain. In BR signaling transduction, nine *BAK1* DEGs showed altered expression (Fig. 3B). BAK1 acts as a co-receptor of BRI1 in BR signal perception and plays an essential role in early signal initiation [52]. BR can promote plant cell elongation and division, effectively regulate plant growth and development, promote stem growth, and increase the number of lateral buds [53]. The differential expression of *BAK1* may affects BR-mediated cell elongation and division processes that regulate stem growth and lateral bud development (Fig. 3B). In Jasmonic acid signaling transduction, JAZ protein (a key negative regulator of JA signal transduction) and MYC2 transcription factor (released after JAZ protein degradation [54]) were down-regulated (Fig. 3B). This down-regulation may alter stress [55] and defense responses [56]. In cytokinin signaling pathway, the down-regulated *A-ARR* gene (a negative regulator [57]), may enhanced cytokinin signaling, further influencing branching. Although direct quantification of endogenous IAA, CTK, ABA, and BR levels was not performed in this study due to experimental constraints, the differential expression of their pathway-related genes provides indirect evidence for the complexity of phytohormone crosstalk. Future studies combining LC-MS/MS-based hormone measurement and genetic validation will help elucidate the underlying regulatory network.

Furthermore, metabolomic analysis further clarified the differential metabolites profile of the mutant *B88*. A total of 54 DAMs were identified, encompassing diterpenes (e.g., GA), flavonoids (e.g., isovitexin), and phenolic acids (e.g., ferulic acid) (Fig. 4A–B, Table S7), all of which are pharmacologically active constituent characteristic of *I. lophanthoides* var. *graciliflorus*. The majority of flavonoid metabolites downregulated, and DEGs involved in hormone pathways exhibited significant correlations with these DAMs associated with the total components (Fig. 4C). The most striking metabolic alteration observed in this study was the extreme downregulation of violanone (Log₂FC = －9.442) (Table S7). Violanone is an isoflavone, a subclass of flavonoid compounds [58]. Flavonoids are widely recognized as key bioactive constituents in numerous medicinal plants, primarily attributed to their prominent antioxidant properties [59]. As one of the major bioactive components of *I. lophanthoides* var. *graciliflorus*, the marked downregulation of violanone may potentially reduce the medicinal quality of this herb. These compositional variations indicate that while the multi-branched mutant exhibits distinct morphological differences that may affect yield (Fig. 1A–E), it also possesses differences in phytochemical contents (Fig. 4B–C). Whether these phytochemical differences influence the medicinal application potential of the multi-branched mutant *B88* requires further investigation in subsequent studies.

Integrated transcriptomic and metabolomic analyses revealed significant enrichment of DEGs and DAMs in diterpenoid biosynthesis pathways, especially GAs biosynthesis pathways (Fig. 5A). GAs plays a key role in regulating plant growth and development [60]. The observed upregulation of bioactive GA_3_ (4.79-fold higher compared to the WT) and differential expression of key biosynthetic enzymes in mutant *B88* implicate GA metabolism as a central regulator of the multi-branched phenotype (Fig. 5). In the GAs synthesis pathway, three key enzyme-encoding genes showed significant differential expression, including 5 important genes encoding enzymes CPS, GA20ox and GA2ox (Fig. 5B). Diterpenoid biosynthesis starts with the formation of the pentacarbon unit isoprene pyrophosphate (IPP) and dimethylallyl pyrophosphate (DMAPP), which are then converted to geranyl geranyl diphosphate (GGPP), the direct precursor for diterpenoid synthesis. In this pathway, the endogenous *CPS* plays a key role, catalyzing the conversion of GGPP to endogenous-cuba pyrophosphate (CDP), a key step in GAs and other diterpenoids biosynthesis. GA20ox enzyme and GA2ox enzyme are located at the final stage of the GAs biosynthesis pathway, respectively. *GA20ox* is mainly responsible for converting GA precursors (e.g., GA_12_ and GA_53_) into bioactive GA forms (e.g., GA_1_ and GA_4_), while GA2ox enzyme controls GAs levels by degrading active GAs or inhibiting its synthesis [61, 62]. In addition, GA2ox can also catalyze the oxidation of specific position (2-β position) in the GAs precursors, converting them into the next intermediate and further affecting the GAs metabolic pathway [58]. In the present study, the differential expression of these key enzyme-encoding genes in *B88* further supported their important roles in regulating GAs biosynthesis and plant growth and development (Fig. 5B). The co-regulation of these differential genes not only affects GAs biosynthesis, but may also have profound effects on plant growth and development processes, including an increased branch number (Fig. 6A). The experimental results of exogenous GA and PAC treatments further supported the significant role of GA in plants (Fig. 6B–F). But it is worth noting that, integrated transcriptomic and metabolomic analyses revealed significantly downregulated expression of key genes in the flavonoid biosynthesis pathway (Fig. 4B–C), sharply reduced levels of the isoflavonoid violanone (Fig. 4B, Table S7), and may lead to a reduction in the accumulation of flavonoid metabolites. These results collectively indicate that the enhanced growth phenotype is coupled with considerable depletion of multiple secondary metabolites (Fig. 4B–C). However, this study has certain limitations. Future research should focus on the long-term stability and heritability of both the enhanced phenotypic traits and the altered phytochemical profile across successive generations (e.g., M_3_, M_4_ plants). Systematic analyses of endogenous phytohormones, phytochemical composition, physiological characteristics, and stress resistance should be incorporated to identify superior genotypes with stable inherited desirable traits, ultimately facilitating the development of elite cultivars with enhanced medicinal quality and agronomic performance.

## Conclusions

The multi-branched mutant *B88* exhibits favorable phenotypic traits including increased branching and plant height, implying high-yield potential. At the molecular level, this study revealed specific genetic variations while maintaining stable chromosome ploidy, hinting at genetic stability and continuity of the species. This mutant enriches the genetic diversity of *I. lophanthoides* var. *graciliflorus* and provides valuable breeding material, lending support to the potential of EMS mutagenesis in medicinal plant improvement. Furthermore, GA may possess comprehensive regulatory effects on plant height, branching patterns, and bioactive compound accumulation in *I. lophanthoides* var. *graciliflorus*, warranting further investigation. The study provides new insights into the effects of EMS mutagenesis on *I. lophanthoides* var. *graciliflorus*, offering preliminary evidence for growth regulation, quality improvement, and germplasm innovation in this species. Future studies should systematically assess the transgenerational stability of trait improvements in advanced generations (e.g., M_3_/M_4_). Multi-dimensional integrated profiling of phytochemical fingerprints, physiological indicators, and stress-response traits will enable the accurate selection of superior genotypes, providing a preliminary basis for breeding elite cultivars with concurrently optimized medicinal and agronomic performance.

## Supplementary Information

Supplementary Material 1: Fig. S1 Chromosome division phase micrograph. The cell division process of the young buds of *I. lophanthoides* var. *graciliflorus*, including interphase (A), prophase (B), metaphase (C), anaphase (D) and telophase (E) under a EX30 light microscope at 100 magnifications. Full-length blots are presented in Additional File 2.

Supplementary Material 2: Fig. S2 AGAR gel electrophoresis of PCR amplification with 24 pairs of primers for SSR polymorphism. “M” is marker, WT is wild-type control, and B88 is the multi-branched mutant. Primer 19 (P19) and primer 21 (P21) had the highest polymorphism rate. Full-length gels are presented in Additional File 3.

Supplementary Material 3: Fig. S3 Real-time quantitative PCR analysis of DEGs.Relative expression levels of (A) *ARF5*, (B) *AUX1*, (C) *GA20oX1D*, (D) *SAUR32*, (E) *BALDH*, (F) *CYP93B2*, (G) *GA20X1*, (H) *YUC8*. The reference genes were shown in Table S3, “ns”, “*” and “**” respectively indicate not significant, *p* < 0.05 and *p* < 0.01.

Supplementary Material 4: Fig. S4 Results of statistical analysis of metabolome data. Principal Component Analysis (PCA) of metabolites identified in CK and *B88* in negative (A) and positive (B) ion mode. Orthogonal partial least squares discriminant analysis (OPLS-DA) analysis in CK and *B88* in positive ion mode (C) and negative (D). Permutation test of OPLS-DA in positive (E) and negative (F) ion mode.

Supplementary Material 5: Table S1 List of polymorphic primer sequences. Table S2 SSR amplified bands analysis results. Amplified bands included all amplified bands of WT and *B88*. The Polymorphic band was the difference between WT and *B88*. Ratio of polymorphism is the ratio of Polymorphic bands to Amplified bands. Table S3 List of primer information for qRT-PCR. Table S4 Statistical table of base information. Table S5 2641 DEGs annotated information in transcriptomics. Table S6 Qualitative and quantitative results of metabolites. Including positive (POS) ion mode and negative (NEG) ion mode. Table S7 Annotation information of 54 DAMs in metabolomics. Including POS ion mode and NEG ion mode. Table S8 Annotation information of KEGG pathway enrichment analysis of metabolomics.

Supplementary Material 6. Full-length blots for Fig. 1F.

Supplementary Material 7. Full-length blots for Supplementary Fig. S1.

Supplementary Material 8. Full-length gels for Supplementary Fig. S2.

Supplementary Material 9. Document S1 Analysis conditions of metabolomics analysis by UPLC-MS/MS. An appropriate amount of sample was added to precooled methanol/acetonitrile/water solution (2:2:1, v/v), vortexed and mixed, sonicated at low temperature for 30 min, left at －20 ℃ for 10min, centrifuged at 14000g at 4 ℃ for 20min. The supernatant was dried under vacuum. For mass spectrometry analysis, 100 μL aqueous acetonitrile solution (acetonitrile: water = 1:1, v/v) was added for dissolution, and then vortexed, centrifuged at 14000 g at 4 ℃ for 15 min, and the supernatant was taken as a sample for UPLC-MS/MS analysis. An aliquot of 2 µL sample was injected into a UHPLC system (Agilent 1290 Infinity III LC System). Metabolites were separated by HILIC chromatographic column (Waters, ACQUITY UPLC BEH Amide 1.7 μm, 2.1 mm×100 mm column); The mobile phase consisted of solvent A, pure water with 25 mM ammonium acetate and 25 mM ammonia water, and solvent B, acetonitrile. The gradient separation started with 95% B and 5% A maintained 0.5 minutes. From 0.5 to 7 min, B changed linearly from 95% to 65%; from 7 to 8 min, B changed linearly from 65% to 40%; from 8 to 9 min, B maintained at 40%; from 9 to 9.1 min, B changed linearly from 40% to 95%; from 9.1 to 12 min, B was maintained at 95%. The flow rate was set as 0.5 mL per minute; The column oven was set to 25 ℃.The primary and secondary spectra of the samples were collected by Triple TOF 6600 system (AB SICEX). The ESI source parameters were set as follows: ion source gas1 (GAS1), 60 psi; ion source gas2 (GAS2),60 psi; curtain gas (CUR), 30 psi; temperature (TEM), 600 ℃; ion spray voltage floating (ISVF), ± 5500V in positive or negative modes, respectively; TOF MS scan m/z range, 60-1000 Da; product ion scan m/z range, 25-1000 Da; TOF MS scan accumulation time 0.20 s/spectra, product ion scan accumulation time 0.05 s/spectra; Secondary mass spectra were obtained with information dependent acquisition (IDA) and in high sensitivity mode with declustering potential (DP), ± 60V in positive or negative modes; Collision Energy, 35 ± 15eV; IDA Settings as follows Exclude isotopes within 4 Da, Candidate ions to monitor percycle, 10.Raw mass spectrometry data were converted to mzML format using ProteoWizard prior to processing with XCMS software for peak alignment, retention time correction, and peak area extraction. Data quality control was performed by removing metabolites with > 50% missing values within any experimental group, followed by KNN-based imputation of remaining null values and elimination of outliers.

## Data Availability

The dataset supporting the conclusions of this article is available in the Genome Sequence Archive repository, GSA: CRA026951 that are publicly accessible at [https://ngdc.cncb.ac.cn/gsa](https:/ngdc.cncb.ac.cn/gsa) .
