# Supplementary figures and images for: A multi-branched EMS mutant of Isodon lophanthoides var. graciliflorus exhibits significant differences in phytohormones and diterpenoids
Source: BMC Plant Biol. 2026 Apr 23;26:1161. doi: 10.1186/s12870-026-08709-1 (PMC13348553; doi:10.1186/s12870-026-08709-1)

A

Interphase

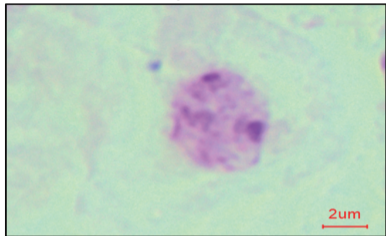

B

Prophase

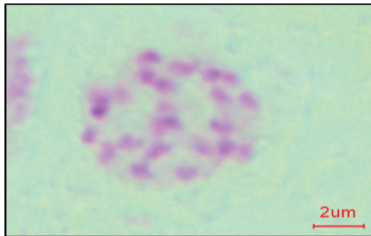

C

Metaphase

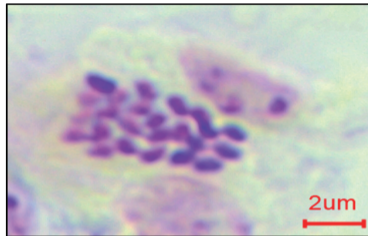

D

Anaphase

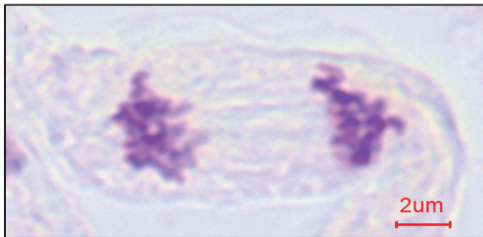

E

Telophase

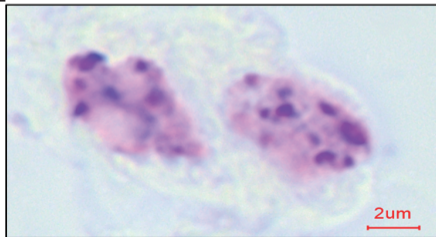

Supplement: Supplementary file 1 — Supplementary Material 1: Fig. S1 Chromosome division phase micrograph. The cell division process of the young buds of I. lophanthoides var. graciliflorus, including interphase (A), prophase (B), metaphase (C), anaphase (D) and telophase (E) under a EX30 light microscope at 100 magnifications. Full-length blots are presented in Additional File 2. [file 12870_2026_8709_MOESM1_ESM.pdf]

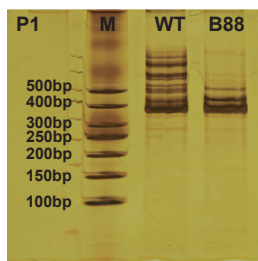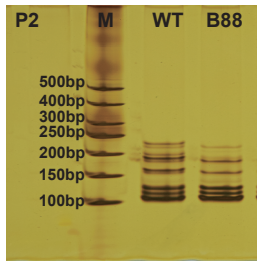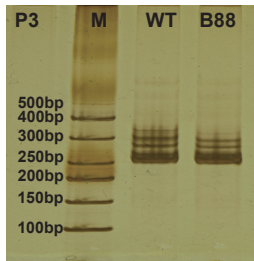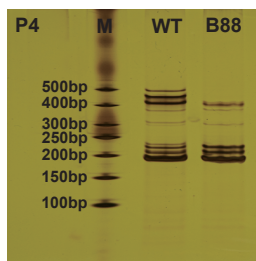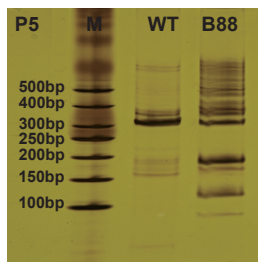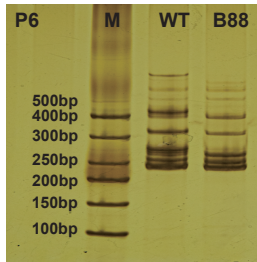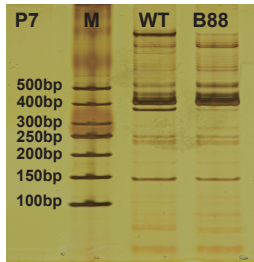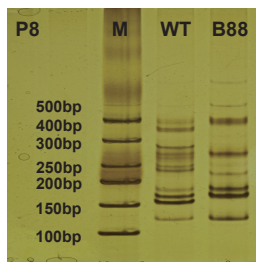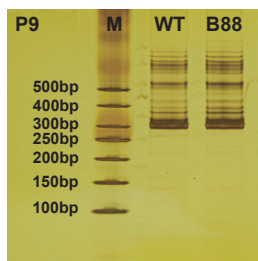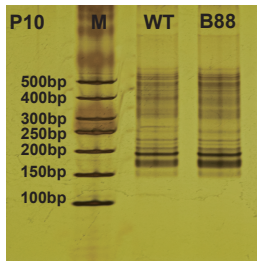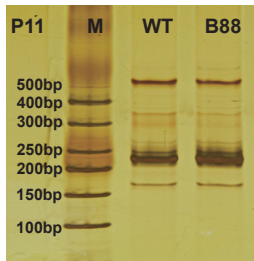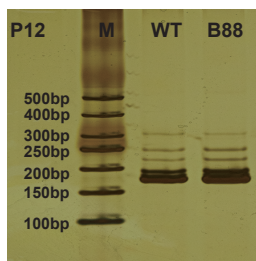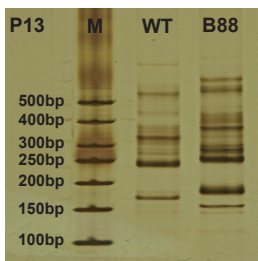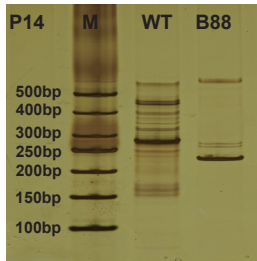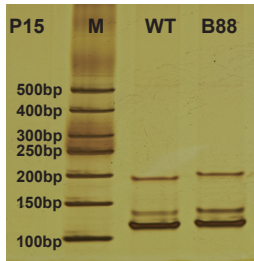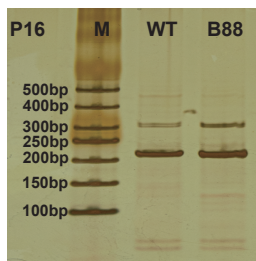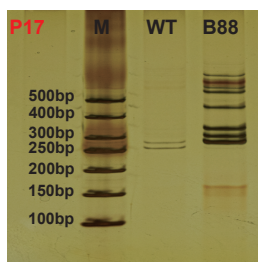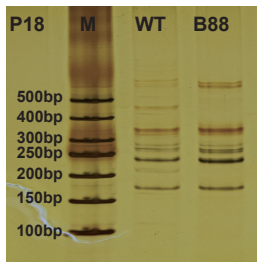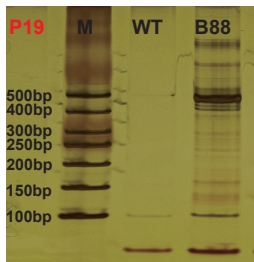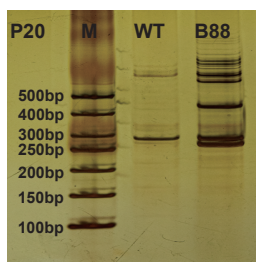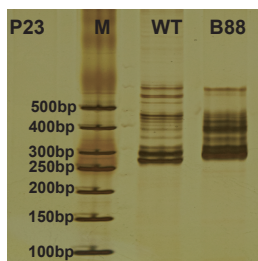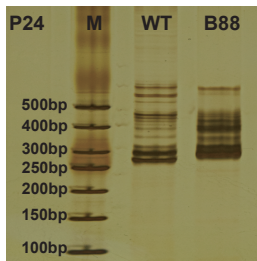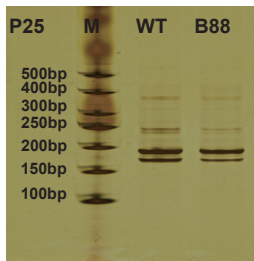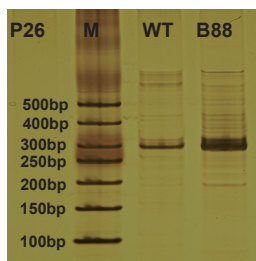

Supplement: Supplementary file 2 — Supplementary Material 2: Fig. S2 AGAR gel electrophoresis of PCR amplification with 24 pairs of primers for SSR polymorphism. “M” is marker, WT is wild-type control, and B88 is the multi-branched mutant. Primer 19 (P19) and primer 21 (P21) had the highest polymorphism rate. Full-length gels are presented in Additional File 3. [file 12870_2026_8709_MOESM2_ESM.pdf]

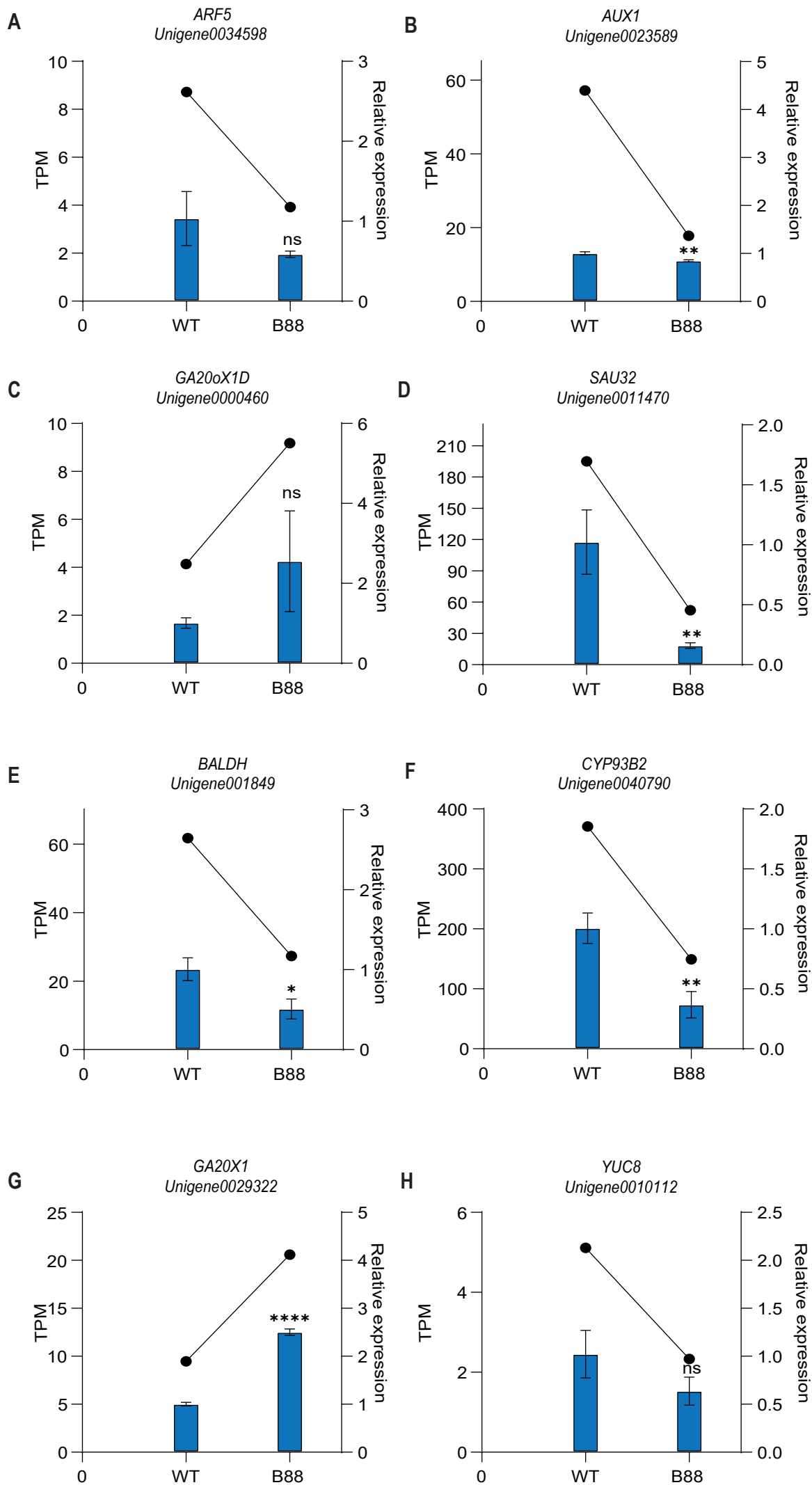

Supplement: Supplementary file 3 — Supplementary Material 3: Fig. S3 Real-time quantitative PCR analysis of DEGs.Relative expression levels of (A) ARF5, (B) AUX1, (C) GA20oX1D, (D) SAUR32, (E) BALDH, (F) CYP93B2, (G) GA20X1, (H) YUC8. The reference genes were shown in Table S3, “ns”, “*” and “**” respectively indicate not significant, p < 0.05 and p < 0.01. [file 12870_2026_8709_MOESM3_ESM.pdf]

A

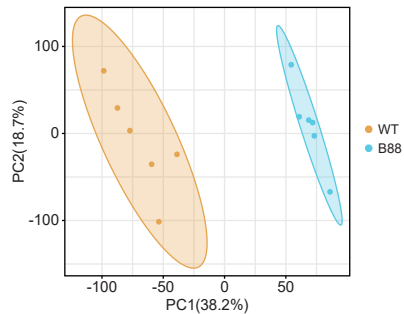

B

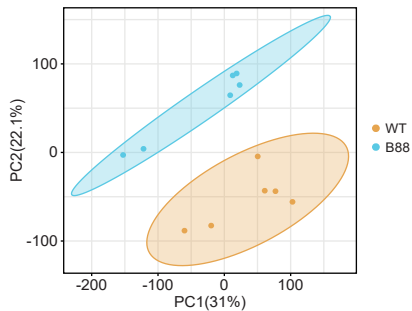

C

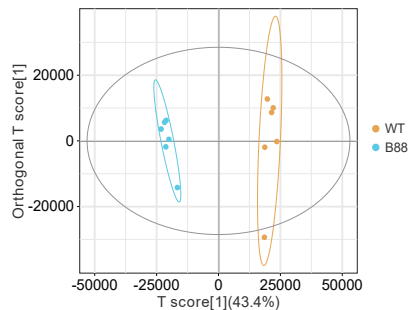

D

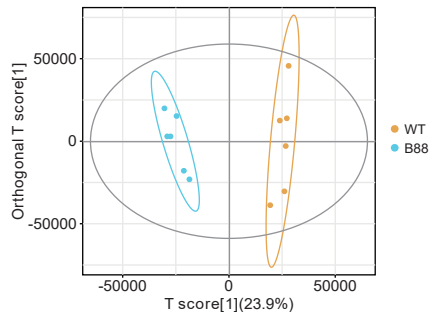

E

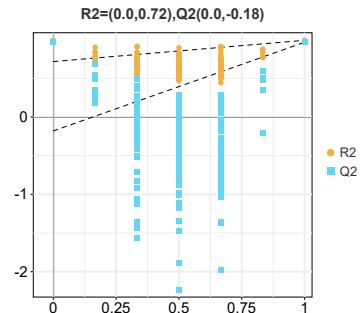

F

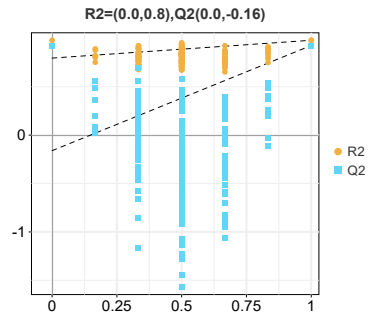

Supplement: Supplementary file 4 — Supplementary Material 4: Fig. S4 Results of statistical analysis of metabolome data. Principal Component Analysis (PCA) of metabolites identified in CK and B88 in negative (A) and positive (B) ion mode. Orthogonal partial least squares discriminant analysis (OPLS-DA) analysis in CK and B88 in positive ion mode (C) and negative (D). Permutation test of OPLS-DA in positive (E) and negative (F) ion mode. [file 12870_2026_8709_MOESM4_ESM.pdf]

Fig.1 F

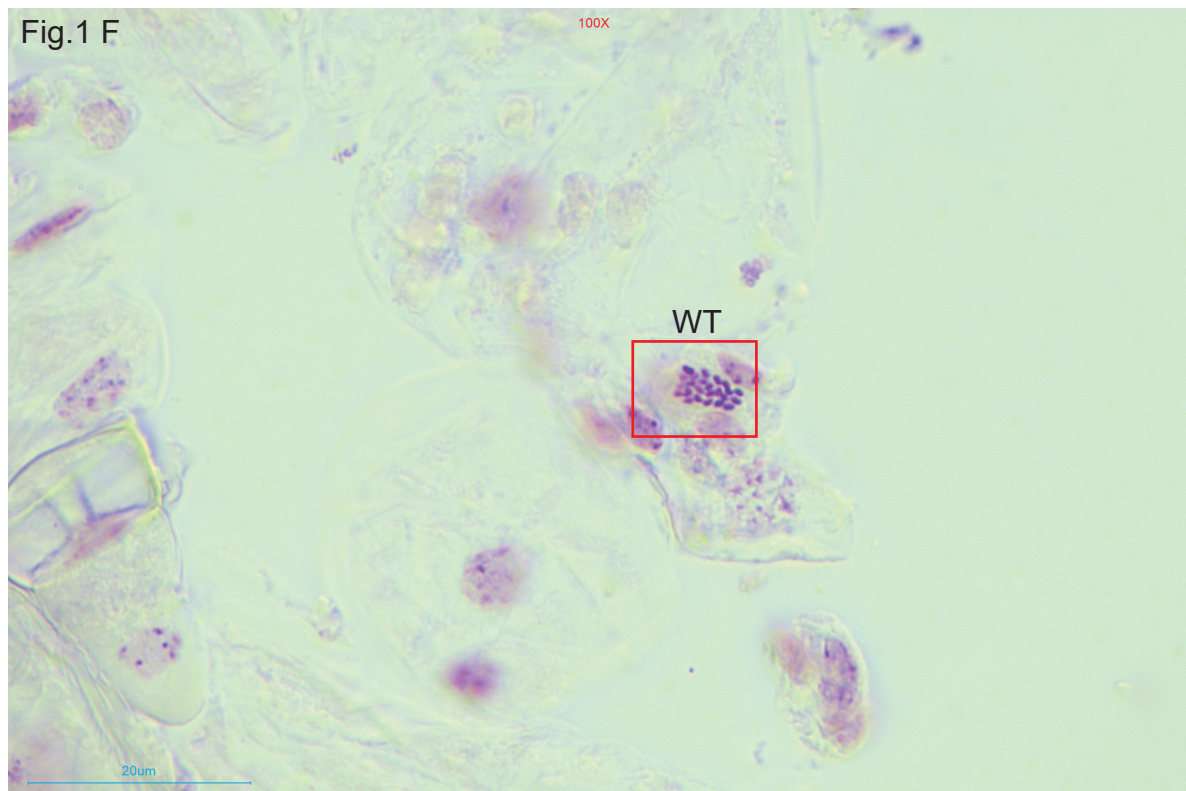

Fig.1 F

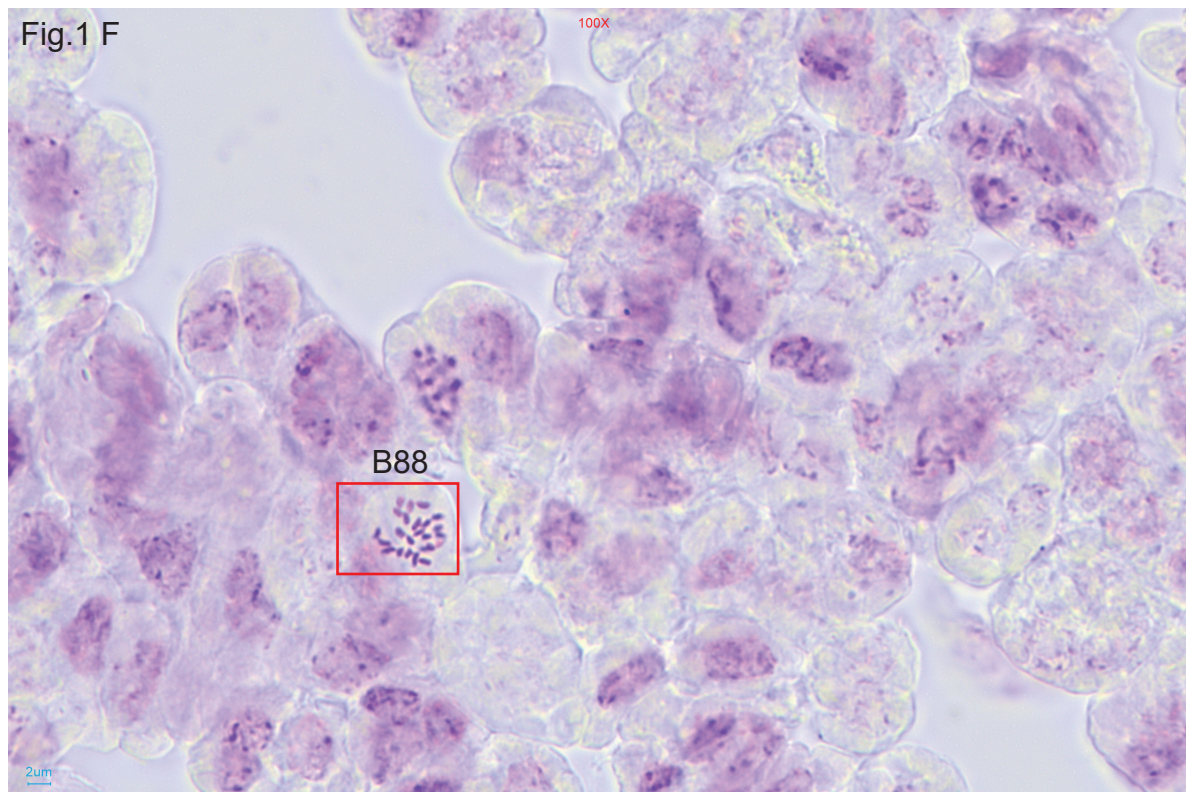

Supplement: Supplementary file 6 — Supplementary Material 6. Full-length blots for Fig. 1F. [file 12870_2026_8709_MOESM6_ESM.pdf]

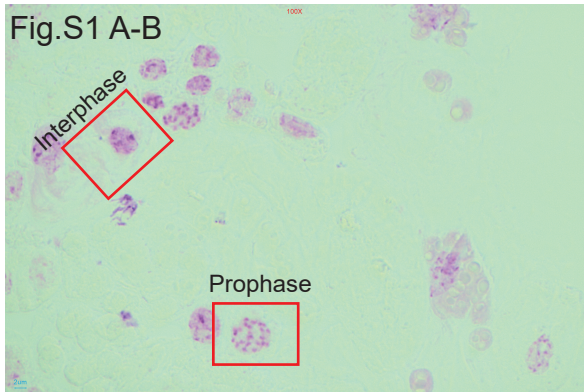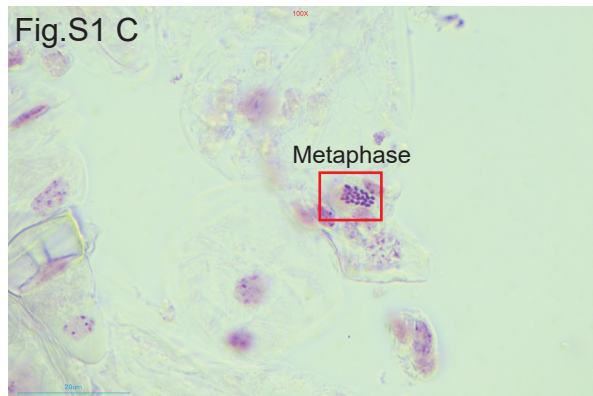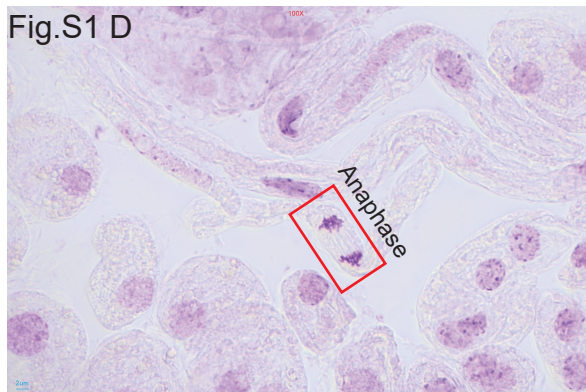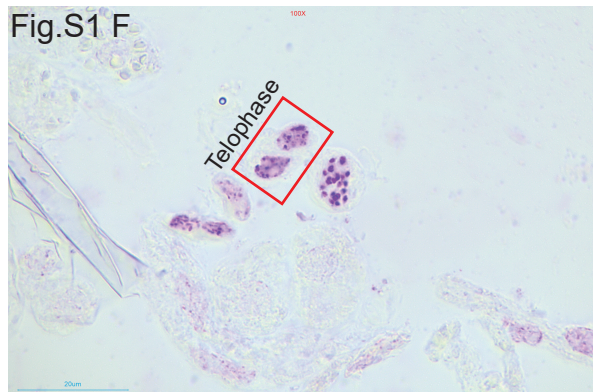

Supplement: Supplementary file 7 — Supplementary Material 7. Full-length blots for Supplementary Fig. S1. [file 12870_2026_8709_MOESM7_ESM.pdf]

**Fig.S2**

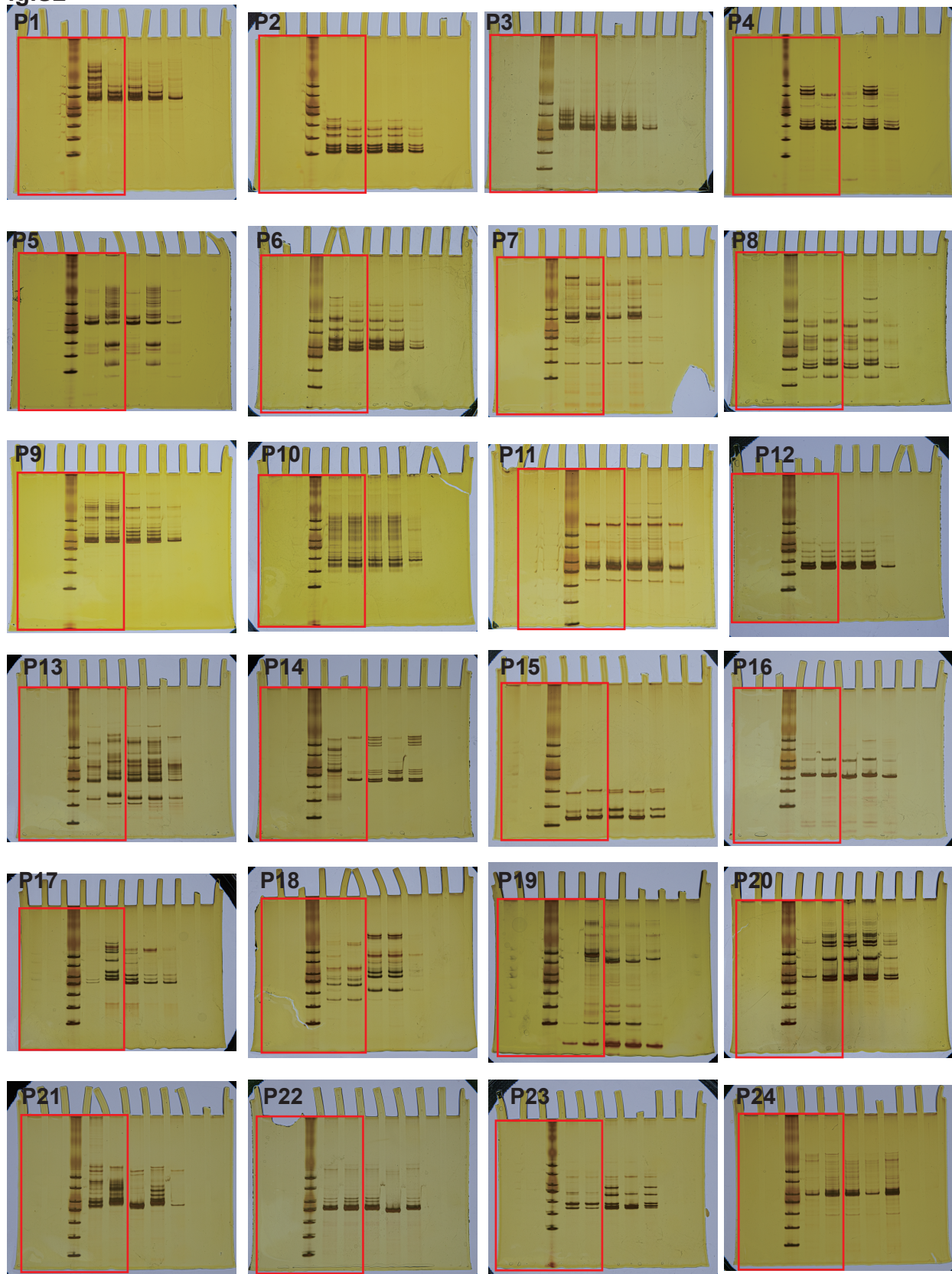

Supplement: Supplementary file 8 — Supplementary Material 8. Full-length gels for Supplementary Fig. S2. [file 12870_2026_8709_MOESM8_ESM.pdf]
