# Supplementary material for: A multi-branched EMS mutant of Isodon lophanthoides var. graciliflorus exhibits significant differences in phytohormones and diterpenoids
Source: BMC Plant Biol. 2026 Apr 23;26:1161. doi: 10.1186/s12870-026-08709-1 (PMC13348553; doi:10.1186/s12870-026-08709-1)
Supplement: Supplementary file 9 — Supplementary Material 9. Document S1 Analysis conditions of metabolomics analysis by UPLC-MS/MS. An appropriate amount of sample was added to precooled methanol/acetonitrile/water solution (2:2:1, v/v), vortexed and mixed, sonicated at low temperature for 30 min, left at －20 ℃ for 10min, centrifuged at 14000g at 4 ℃ for 20min. The supernatant was dried under vacuum. For mass spectrometry analysis, 100 μL aqueous acetonitrile solution (acetonitrile: water = 1:1, v/v) was added for dissolution, and then vortexed, centrifuged at 14000 g at 4 ℃ for 15 min, and the supernatant was taken as a sample for UPLC-MS/MS analysis. An aliquot of 2 µL sample was injected into a UHPLC system (Agilent 1290 Infinity III LC System). Metabolites were separated by HILIC chromatographic column (Waters, ACQUITY UPLC BEH Amide 1.7 μm, 2.1 mm×100 mm column); The mobile phase consisted of solvent A, pure water with 25 mM ammonium acetate and 25 mM ammonia water, and solvent B, acetonitrile. The gradient separation started with 95% B and 5% A maintained 0.5 minutes. From 0.5 to 7 min, B changed linearly from 95% to 65%; from 7 to 8 min, B changed linearly from 65% to 40%; from 8 to 9 min, B maintained at 40%; from 9 to 9.1 min, B changed linearly from 40% to 95%; from 9.1 to 12 min, B was maintained at 95%. The flow rate was set as 0.5 mL per minute; The column oven was set to 25 ℃.The primary and secondary spectra of the samples were collected by Triple TOF 6600 system (AB SICEX). The ESI source parameters were set as follows: ion source gas1 (GAS1), 60 psi; ion source gas2 (GAS2),60 psi; curtain gas (CUR), 30 psi; temperature (TEM), 600 ℃; ion spray voltage floating (ISVF), ± 5500V in positive or negative modes, respectively; TOF MS scan m/z range, 60-1000 Da; product ion scan m/z range, 25-1000 Da; TOF MS scan accumulation time 0.20 s/spectra, product ion scan accumulation time 0.05 s/spectra; Secondary mass spectra were obtained with information dependent acquisition (IDA) and in high se [file 12870_2026_8709_MOESM9_ESM.docx]

**Document S1 Analysis conditions of metabolomics analysis by UPLC-MS/MS**

An appropriate amount of sample was added to precooled methanol/acetonitrile/water solution (2:2:1, v/v), vortexed and mixed, sonicated at low temperature for 30 min, left at －20 ℃ for 10min, centrifuged at 14000g at 4 ℃ for 20min. The supernatant was dried under vacuum. For mass spectrometry analysis, 100 μL aqueous acetonitrile solution (acetonitrile: water = 1:1, v/v) was added for dissolution, and then vortexed, centrifuged at 14000 g at 4 ℃ for 15 min, and the supernatant was taken as a sample for UPLC-MS/MS analysis. An aliquot of 2 µL sample was injected into a UHPLC system (Agilent 1290 Infinity III LC System). Metabolites were separated by HILIC chromatographic column (Waters, ACQUITY UPLC BEH Amide 1.7 μm, 2.1 mm×100 mm column); The mobile phase consisted of solvent A, pure water with 25 mM ammonium acetate and 25 mM ammonia water, and solvent B, acetonitrile. The gradient separation started with 95% B and 5% A maintained 0.5 minutes. From 0.5 to 7 min, B changed linearly from 95% to 65%; from 7 to 8 min, B changed linearly from 65% to 40%; from 8 to 9 min, B maintained at 40%; from 9 to 9.1 min, B changed linearly from 40% to 95%; from 9.1 to 12 min, B was maintained at 95%. The flow rate was set as 0.5 mL per minute; The column oven was set to 25 ℃.

The primary and secondary spectra of the samples were collected by Triple TOF 6600 system (AB SICEX). The ESI source parameters were set as follows: ion source gas1 (GAS1), 60 psi; ion source gas2 (GAS2),60 psi; curtain gas (CUR), 30 psi; temperature (TEM), 600 ℃; ion spray voltage floating (ISVF), ± 5500V in positive or negative modes, respectively; TOF MS scan m/z range, 60-1000 Da; product ion scan m/z range, 25-1000 Da; TOF MS scan accumulation time 0.20 s/spectra, product ion scan accumulation time 0.05 s/spectra; Secondary mass spectra were obtained with information dependent acquisition (IDA) and in high sensitivity mode with declustering potential (DP), ± 60V in positive or negative modes; Collision Energy, 35 ± 15eV; IDA Settings as follows Exclude isotopes within 4 Da, Candidate ions to monitor percycle, 10.

Raw mass spectrometry data were converted to mzML format using ProteoWizard prior to processing with XCMS software for peak alignment, retention time correction, and peak area extraction. Data quality control was performed by removing metabolites with > 50% missing values within any experimental group, followed by KNN-based imputation of remaining null values and elimination of outliers.
